# Multi-scale characterization of glaucophane from Chiavolino (Biella, Italy): implications for international regulations on elongate mineral particles

**DOI:** 10.5194/ejm-33-77-2021

**Published:** 2021-02-09

**Authors:** Ruggero Vigliaturo, Sabrina M. Elkassas, Giancarlo Della Ventura, Günther J. Redhammer, Francisco Ruiz-Zepeda, Michael J. O’Shea, Goran Dražić, Reto Gieré

**Affiliations:** 1 Department of Earth and Environmental Science, University of Pennsylvania, 240 S. 33rd Street, Hayden Hall, Philadelphia, PA, USA; 2 Department of Earth, Atmospheric, and Planetary Sciences, Massachusetts Institute of Technology, 77 Massachusetts Avenue, Cambridge, MA, USA; 3 Department of Marine Chemistry and Geochemistry, Woods Hole Oceanographic Institution, Woods Hole, MA, USA; 4 Department of Geological Sciences, University of Roma Tre, Rome, Italy; 5 INFN-Istituto Nazionale di Fisica Nucleare, Frascati, Rome, Italy; 6 Department of Materials Science and Physics, University of Salzburg, Salzburg, Austria; 7 Department of Physics and Chemistry of Materials, Institute of Metals and Technology, Lepi pot 11, Ljubljana, Slovenia; 8 Department of Materials Chemistry, National Institute of Chemistry, Hajdrihova 19, Ljubljana, Slovenia; 9 Center of Excellence in Environmental Toxicology, University of Pennsylvania, Philadelphia, PA, USA

## Abstract

In this paper, we present the results of a multi-analytical characterization of a glaucophane sample collected in the Piedmont region of northwestern Italy. Investigation methods included optical microscopy, powder X-ray diffraction, Fourier-transform infrared spectroscopy, μ-Raman spectroscopy, Mössbauer spectroscopy, electron probe microanalysis, environmental scanning electron microscopy and energy-dispersive X-ray spectroscopy, and scanning/transmission electron microscopy combined with energy-dispersive X-ray spectroscopy and electron energy-loss spectroscopy. In addition to the crystal–chemical characterization of the sample from the mesoscale to the near-atomic scale, we have also conducted an extended study on the morphology and dimensions of the mineral particles. The main finding is that studying the same particle population at different magnifications yields different results for mineral habit, dimensions, and dimensional distributions. As glaucophane may occur as an elongate mineral particle (e.g., asbestiform glaucophane occurrences in California and Nevada), the observed discrepancies therefore need to be considered when assessing potential breathability of such particles, with implications for future regulations on elongate mineral particles. While the sample preparation and particle counting methods are not directly investigated in this work, our findings suggest that different magnifications should be used when characterizing an elongate mineral particle population, irrespective of whether or not it contains asbestiform material. These results further reveal the need for developing improved regulation for elongate mineral particles. We thus propose a simple methodology to merge the datasets collected at different magnifications to provide a more complete description and a better risk evaluation of the studied particle population.

## 1 Introduction

Glaucophane is a silicate mineral that belongs to the sodic amphibole group (Hawthorne et al., 2012). The root of the name “glaucophane” is the Greek *glaukos*, which means bluish green, and *phainesthai*, which means to appear. The type locality of glaucophane is on the island of Syros (Cyclades, Greece), where metamorphic rocks including blueschists, metapelites, and glaucophane-bearing marbles (Ridley, 1981) underwent two distinct Eocene eclogite–blueschist facies events: the first occurring at 470–520 °C and 14–18 kb, and the second at up to 460 °C and 14 kb (Lister and Raouzaious, 1996). Historically, glaucophane was used as natural blue pigment during the Minoan period, especially in Santorini and Knossos, Crete. Its use was particularly widespread during the “Last Palace” period, beginning in the Middle Minoan III in 1700 BCE (Filippakis et al., 1976; Profi et al., 1976). Glaucophane represents one of the main amphiboles in blueschist-facies metamorphic rocks, where it plays a key role in defining conditions of medium-to high-pressure and low-temperature metamorphism (Jenkins et al., 2013). Glaucophane is associated with basaltic and gabbroic protoliths, in which it crystallizes as a result of subduction.

Glaucophane crystallizes in the monoclinic system and is classified in the sodium amphibole group, defined by ^B^(Na+Li)*/*ΣB≥0.75, ^B^Na*/*ΣB> ^B^Li*/*ΣB (Hawthorne et al., 2012). Its end-member formula is Na_2_[Mg_3_Al_2_]Si_8_O_22_(OH)_2_ but often forms a solid solution with riebeckite ${\text{Na}}_{2}\left[{\text{Fe}}_{3}^{2+}{\text{Fe}}_{2}^{3+}\right]{\text{Si}}_{8}{\text{O}}_{22}{\left(\text{OH}\right)}_{2}$, which, in its asbestiform morphology, is known as crocidolite or blue asbestos. Glaucophane may occur as an elongate mineral particle, and specifically with an asbestiform habit, or it may host asbestiform riebeckite (crocidolite) particles (Erskine and Bailey, 2018; Bailey, 2020a, b). Furthermore, it is likely that the particulate matter released into the atmosphere from glaucophane- and/or riebeckite-bearing rocks during excavation and mining activities (Bailey, 2020a, b) is a mixture of particles. This mixture contains particles that range in morphology from fibrous to non-fibrous (Oberdörster and Graham, 2018) and might or might not be respirable. There is no general agreement yet whether or not nano-sized mineral particles (usually defined has having at least one dimension <100 nm) with certain dimensions and aspect ratios should be considered as elongate mineral particles (EMPs) (Oberdörster and Graham, 2018). The French ANSES (2015) report, however, already takes into account the nano-sized portion of EMPs by defining an EMP as any particle with length/width (*L/w*) >3. The nano-sized portion of EMPs is ubiquitous; it can be found in the environment (e.g., Petriglieri et al., 2020), where humans may be exposed to industrially processed particles (e.g., Vigliaturo et al., 2016), and in the lungs of rats that live in urban areas with a heavy presence of asbestos released from industrial facilities (e.g., Ardizzone et al., 2014). In this respect, multiple literature sources suggest that counting and recording the presence or absence of nano-sized EMPs could be the best choice in terms of regulation and public health protection (Le Bouffant et al., 1987; Morrow, 1988; Donaldson et al., 2010; Oberdörster and Graham, 2018).

The term “EMP”, which should substitute the term “fiber” (NIOSH, 2011), refers to a mineral particle that exhibits an aspect ratio (*L/w*) of ≥ 3 : 1, has an *L*>5 μm, and may include either an asbestiform or a non-asbestiform habit (NIOSH, 2011). The replacement of the term “fiber” with the term “EMP” was made specifically to include both the asbestiform and the non-asbestiform habits, which meet the dimensional criteria specified by NIOSH (2011). For clarity, in this paper, we will use the term EMP as defined by NIOSH (2011), whereas the term “fiber” will be used as defined in Belluso et al. (2017), i.e., for elongated particles with uniform parallel sides and geometrical faces exhibiting *L/w* ≥ 3, *L* ≥ 5 μm, and *w* ≤ 3 μm. If the term “fiber” will be used to mineralogically identify a habit (as described in Zoltai, 1981; Veblen and Wylie, 1993), this will be specified in the text.

In this paper, we characterize a non-asbestiform glaucophane sample collected in Chiavolino (province of Biella), located in the Italian Piedmont region. Following a brief geological description of the sample locality, we report on a systematic characterization of the collected sample. The analysis of the sample was conducted by applying optical microscopy (OM), powder X-ray diffraction (PXRD), electron probe micro-analysis (EPMA), Fourier-transform infrared spectroscopy (FTIR), μ-Raman spectroscopy, field-emission gun environmental scanning electron microscopy (FEG-ESEM), transmission electron microscopy with selected area electron diffraction (TEM-SAED, JEOL 2010F), high-resolution TEM (HR-TEM, JEOL F200), and aberration-corrected scanning transmission electron microscopy (accorrected JEOL ARM 200CF STEM; acSTEM), combined with energy-dispersive X-ray spectrometry (EDXS) and dual-range electron energy-loss spectroscopy (Dual-EELS). The simulated STEM image was calculated based on the atomic model for glaucophane and the microscope parameters using the quantitative STEM (QSTEM) code with a multi-slice method and frozen phonon approximation. We also conducted an extended dimensional characterization of the glaucophane by using OM, FEG-ESEM, and acSTEM. This study concludes with an acSTEM tomographic three-dimensional (3-D) reconstruction of a particle to show how the orientation of the observed material may change the size classification and the habit of the particle, and its measured dimension in a 2-D projection.

In short, we demonstrate how an extended dimensional study using multiple magnifications allows for a better and more complete description of the real size and dimensional distributions of a given particle population. The multi-magnification characterization of a mineral particle population avoids underestimating the number of particles that are classified by different dimensional parameters and different shape or morphology, and that might be dangerous to public health and in industrial settings.

## 2 Geological setting

**Figure 1.**
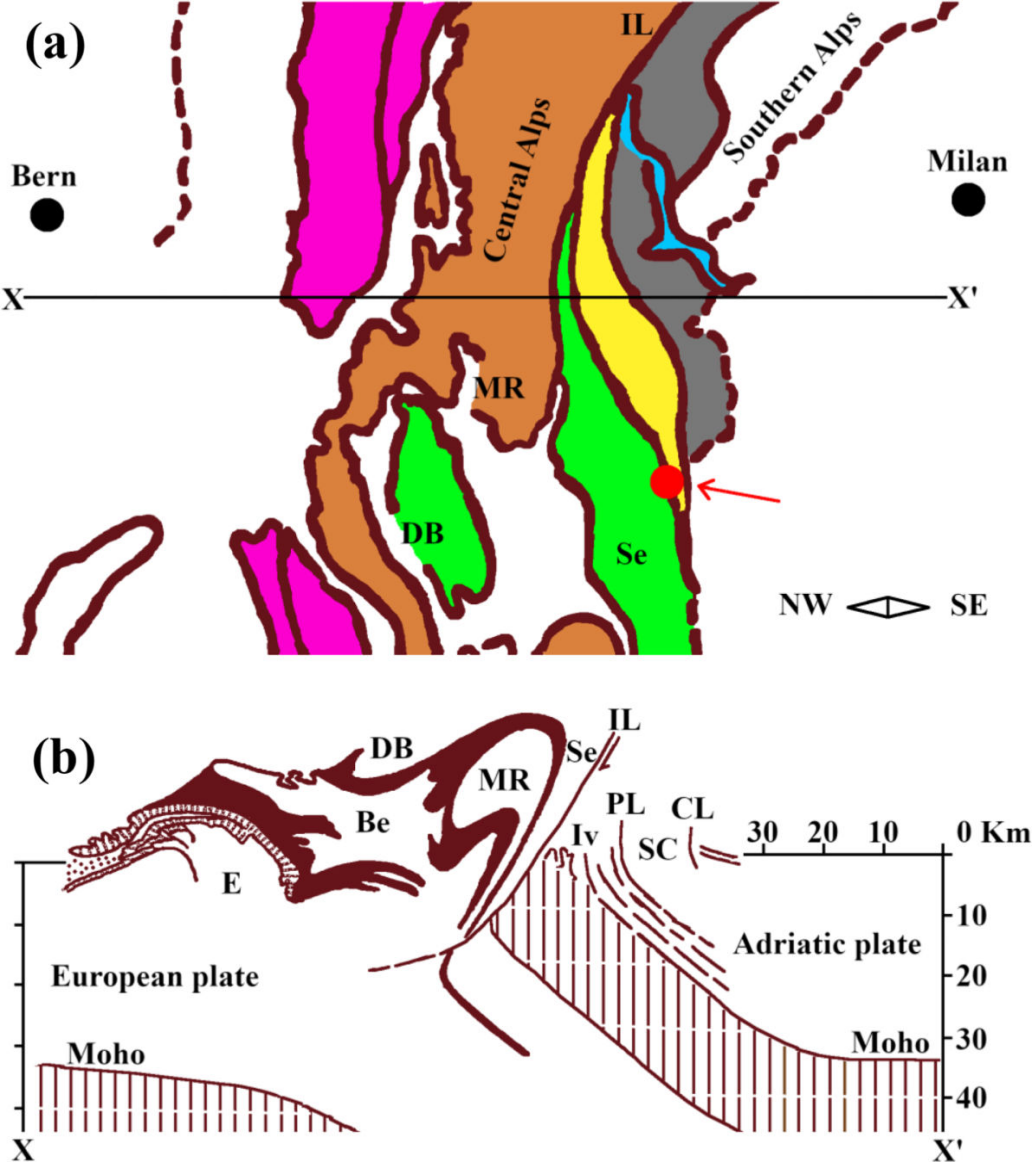
**(a)** Tectonic setting of the sample locality, digitally re-drawn and modified after Zingg (1990). The *X*–*X*′ line represents the profile section shown in panel **(b)**. In panel **(a)**, the purple area represents the external massifs, the light brown areas are the Penninic basement, the green area shows the Austroalpine basement, and the orange area is the South Alpine basement. The yellow area is the Ivrea zone, and the light blue area is Lake Maggiore. Sample locality is indicated by the red dot and red arrow. **(b)** Cross-section through the Western Alps, digitally re-drawn after Zingg (1990). E: external massifs, Be: Bernhard nappe, DB: Dent Blanche nappe, MR: Monte Rosa nappe, Se: Sesia zone, Iv: Ivrea zone, IL: Insubric line, PL: Pogallo line, SC: Strona–Ceneri zone; CL: Cremosina line.

Glaucophane is found globally and is associated with mountain-building events and past subduction zones. The most notable localities include the California Coast Ranges, USA; Kodiak Island, Alaska, USA; and St. Marcel, Val d’Aosta, and Piollore (Biella), Piedmont, Italy (Evans and Brown, 1986). The geological setting of the Italian Piedmont region, where our sample was found, is the result of complex geodynamic processes. Starting in the Late Cretaceous, two continental margins (the paleo-European and the paleoAdriatic) gradually converged and caused subduction of the oceanic domains (Piedmont–Liguria and Valais), which were entrapped between the continental plates (Piana et al., 2017). In the middle to late Eocene, the margins began colliding to form the Alps–Apennines (see Fig. 1a–b and Zingg, 1990), and as a result, Piedmont contains an almost complete cross-section of the crust (Piana et al., 2017).

The Biella province is situated in an area of dynamic metamorphism within the Sesia–Lanzo and the Ivrea–Verbano zones (Fig. 1b and Compagnoni and Schmid, 2014). The Sesia–Lanzo zone (SLZ) is a wide, preserved portion of the continental crust. During the early Alpine subduction in the Western Alps, the section was exposed to eclogite-facies conditions and was exhumed before the collision of the paleo-European and the paleo-Adriatic continental margins (Spalla et al., 1996; Cantù et al., 2016). The Ivrea–Verbano zone, part of the Southern Alps, is an exposed section of the lower crust and upper mantle, which underwent extensive high-pressure and high-temperature metamorphism (from granulite grade in the NW to high-temperature amphibolite grade in the SE; Quick et al., 1992). The unit in which the studied sample was found is known as the Eclogitic Micaschist Complex (EMC). This complex contains glaucophane schists, which represent former eclogites that have undergone retrograde metamorphism (Reinsch, 1979). The main mineralogical components of this unit are omphacite, lawsonite or epidote, garnet, and glaucophane (Bezacier et al., 2010).

## 3 Experimental

### 3.1 Sample

**Figure 2.**
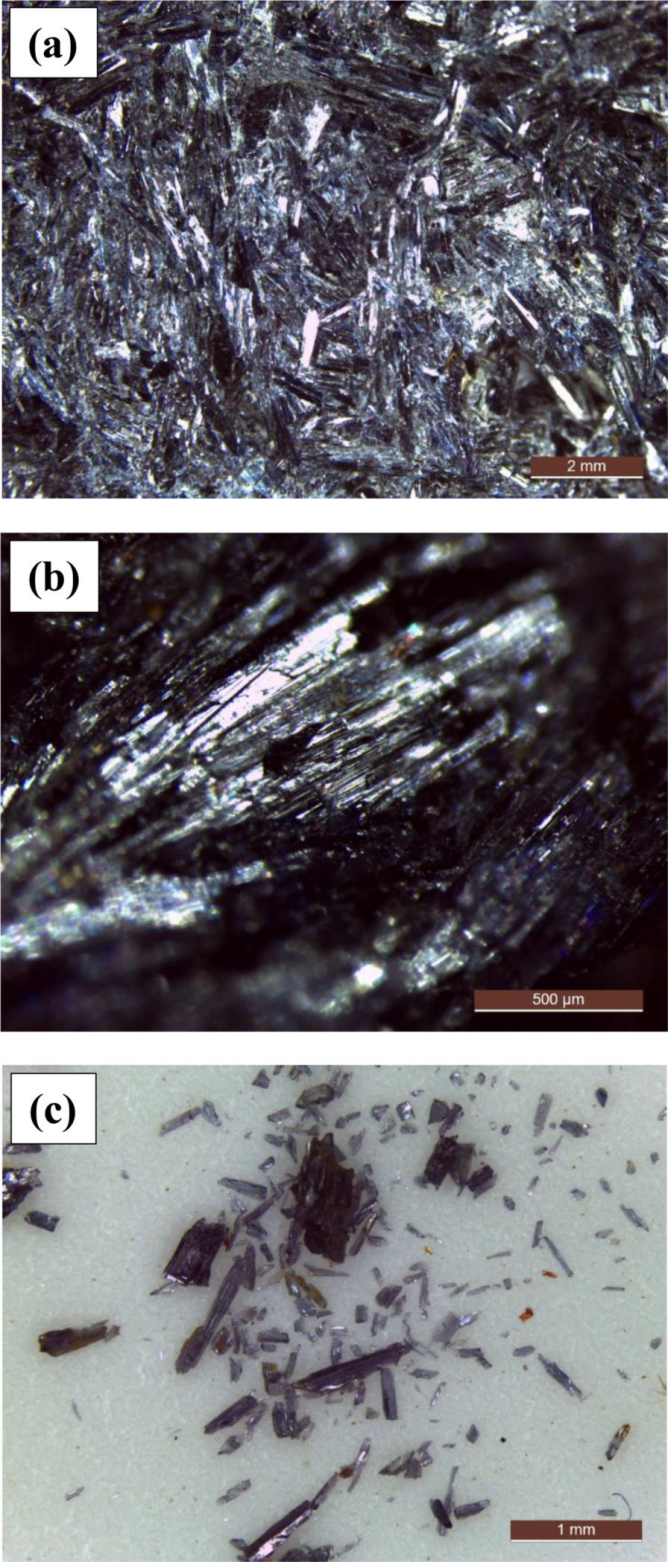
Optical microscopy pictures of the glaucophane sample. **(a)** A mix of columnar, acicular, and prismatic randomly oriented crystals; **(b)** parallel columnar crystals; and (c) crystals with various morphologies detached from the main rock by handling or scratching.

**Figure A1.**
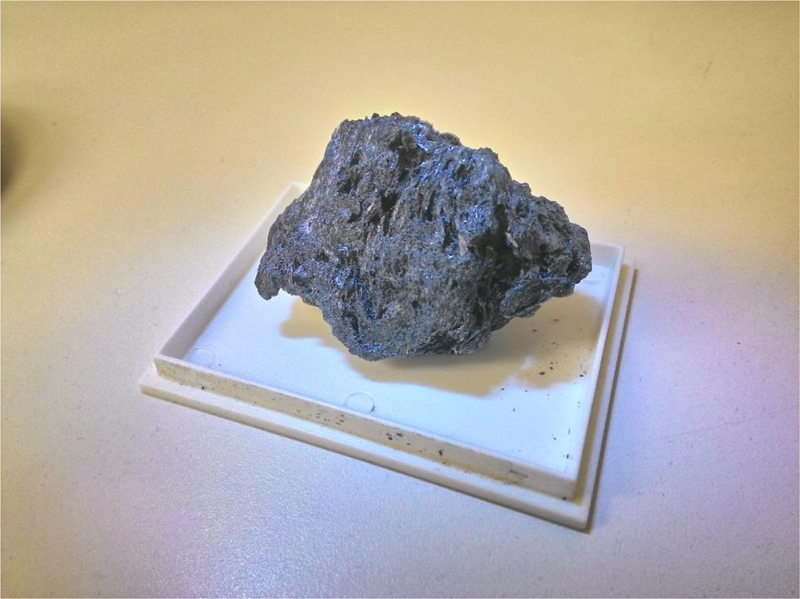
Hand sample as collected in the Chiavolino hamlet (Italy) before any manipulation. The size of the sample is about 5×2 cm.

The hand specimen analyzed here was collected from an outcrop near the trail to an abandoned quarry along Rio Oremo, upstream of Chiavolino hamlet, which is located in the province of Biella, a part of the Italian Piedmont region. The area in which the sample was found is characterized by the presence of a grey, dark-blue-shaded amphibole-rich rock with carbonate veins. The hand sample (~5×2 cm), shown in the Appendix A (Fig. A1), contains mostly glaucophane (Fig. 2), which is accompanied by minor grey-greenish micas, carbonate veins, and some centimeter-sized pyrite cubes (Mottana et al., 1981; Boscardin and Orlandi, 1984).

### 3.2 OM

**Figure B1.**
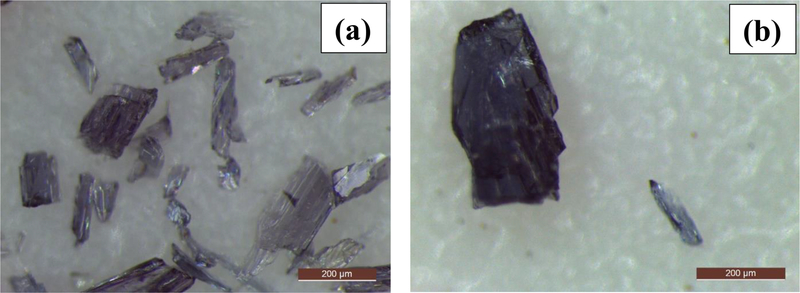
**(a, b)** Fragments detached from the hand sample by applying finger pressure and by scraping with tongs. The images were recorded at 10× in OM.

The hand sample (Fig. A1) was examined as received and broken into small fragments (~3mm across) using tongs and/or finger pressure. These fragments (Figs. 2c, B1) were studied without further preparation using a Leica M165 C stereomicroscope equipped with a Leica IC80 D camera and an LED illumination system.

### 3.3 PXRD

The powder X-Ray diffraction data were collected on glaucophane powders using a Scintag X1 diffractometer (Thermo Fisher, Waltham, MA, USA) equipped with a Cu–*Kα*_1_ radiation source (*λ* = 1.54055 Å, 40 mA, 45 kV), fixed divergence slits, and a Peltier-cooled Si (Li) detector with a resolution of <200 eV. A divergent slit width of 2mm and a scatter-slit width of 4mm were used for the incoming beam, whereas a receiving slit width of 0.5mm and scatter-slit width of 0.2mm were used for the diffracted beam. Data were collected in step-scan mode in the 2–70° 2*θ* range, with a step size of 0.05° 2*θ*, and a counting time of 3 s per step.

### 3.4 FTIR spectroscopy

The FTIR spectra of the glaucophane powders were collected at Roma Tre University using a Nicolet iS50 spectrometer, equipped with a Globar source, a potassium bromide (KBr) beam splitter, and a deuterated triglycine sulfate (DTGS) detector. The powdered samples were prepared as KBr pellets, using 5 : 150 and 0.5 : 150 mg sample-to-KBr ratios for the 3000–4000 and <1200 cm^−1^ regions, respectively. Single-crystal FTIR spectra in the OH-stretching mediuminfrared (MIR) region were collected with unpolarized light, using a Bruker Hyperion 3000 microscope equipped with a mercury–cadmium–telluride (MCT) detector and a KBr beam splitter at Istituto Nazionale di Fisica Nucleare (INFN, Frascati, Rome).

### 3.5 μ-Raman spectroscopy

Unpolarized μ-Raman spectra of untreated samples were collected using a confocal Jasco NRS 5500 microscope at INFN (Frascati) equipped with a multi-channel charge-coupled device (CCD) detector and a fully automated stage. Raman spectra were excited with a 532 nm laser, an integration time of 5 s per scan, averaging 20 scans, with a 5× objective; the laser power was set to 20mW. The wavenumber accuracy was ±0.5 cm^−1^, and the spectral resolution was 1 cm^−1^.

### 3.6 Mössbauer spectroscopy

Transmission ^57^Fe Mössbauer data were acquired on glaucophane powders at room temperature using an apparatus in horizontal arrangement (^57^Fe Co*/*Rh single-line thin source, constant acceleration mode with symmetric triangular velocity shape, multi-channel analyzer with 1024 channels, regular velocity calibration against metallic Fe). Data were evaluated using the RECOIL program suite. All spectra were corrected for thickness effects and then processed using the full static hyperfine interaction Hamiltonian analysis with Lorentzian-shaped doublets. Details of the sample preparation and data handling can be found in Redhammer and Roth (2002).

### 3.7 EPMA

The electron probe micro-analysis data for untreated individual crystals (embedded in resin and polished) were collected by wavelength-dispersive X-ray spectroscopy (WDXS) techniques using a Cameca SX50 electron microprobe (Cameca, Gennevilliers, France) at Centro di Studi per il Quaternario e l’Evoluzione ambientale – CNR, Rome. Analytical conditions were 15 keV accelerating voltage, 20 nA beam current, 5 μm beam size, and 100 s counting time. The data were processed by using the ZAF4/FLS software (standard version) by Link Analytical (Oxford, UK). Compositions were determined relative to the following natural and synthetic standards: diopside (Si, Mg, Ca), metal oxide (Ti, Fe, Mn), orthoclase (Al, K), albite (Na), and fluorite (F).

### 3.8 FEG-ESEM EDXS

A FEI Quanta 600 FEG Mark II field-emission SEM equipped with a Bruker Quantax Silicon Drift Detector was used at the Singh Center for Nanotechnology, University of Pennsylvania, to obtain detailed information on the studied glaucophane. To prepare the sample for electron microscopy (EM) and to obtain proper particle dimensions, it was subjected to gentle hand grinding by pestle and mortar in 2-propanol. Hand grinding was done for 5 min, in agreement with previous investigations of amphiboles (Ministerial Decree, 1994; Vigliaturo et al., 2018). This procedure yields a granulometry that is similar to the one of the Union for International Cancer Control (UICC) asbestos standards at a magnification of 1000× and 2000× but without compromising the crystal structure of the amphibole particles (Ministerial Decree, 1994). The ground material was then transferred into an Eppendorf tube and brought into 2-propanol suspension for 5 min of low-power sonication to promote dispersion of the suspended particles.

Part of the suspension was transferred onto a 12.7mm SEM stub covered by conductive carbon tabs. The SEM was set to environmental mode, with a voltage of 15 kV and a chamber pressure of 0.38 torr, and operated in secondary electron (SE) mode. Variable spot dimension was used to optimize imaging (sizes 3 and 6).

Additionally, in order to complement the morphological study, we have prepared a sample on a 70° inclined SEM stub, which allows operators to observe morphologies that are not usually visible on a flat stub (e.g., the (002) plane). The size-distribution and morphological studies were conducted under the same conditions but on flat stubs and at a magnification of 200× and 2000×.

### 3.9 Aberration-corrected S/TEM EDXS and Dual-EELS study

A droplet of the same suspension prepared for SEM was transferred to each of three TEM copper grids (one holey carbon grid, two lacey carbon support films – SPI) after quick vortexing in a mixer (Corning™ LSE™ vortex mixer) to enhance particle dispersion. The holey carbon grid sample was used for dimensional measurements and for high-resolution TEM, whereas one of the lacey carbon grid samples was used for EELS and HR-TEM. The second lacey carbon grid was cut halfway in order to perform acSTEM tomography.

An acSTEM, model ARM 200 CF equipped with a high-brightness cold-field emission gun (CFEG) operating at 80 kV, an energy-dispersive X-ray spectroscopy (EDXS) system (Centurio 100mm^2^, JEOL), and an energy filter (QuantumGIF, Gatan, USA) were used at the National Institute of Chemistry, Slovenia. The Gatan cryotransfer holder was employed to perform HR-STEM at LN_2_ temperature in order to reduce the effects of radiation damage on the sample during image acquisition.

The dimensional measurements of particle width (*w*) and length (*L*) were performed in STEM bright-field (BF) mode. Morphological observations of the sample were made in TEM mode, in STEM BF mode, and in medium-angle annular dark-field (MAADF) mode. To further augment the morphological description of the specimen, electron tomography and 3-D reconstruction were performed on a selected particle. The specimen was rotated around the JEOL tomography holder *α* axis for a range of 120° with a step of 1°. For each step, a STEM BF and a MAADF image were collected, and the 3-D reconstruction was performed using the JEOL software TEMography™.

Dual-EELS was performed at the grain boundary of the particles on 10 different square areas of 625 nm^2^ each (Vigliaturo et al., 2019). The core-loss region was registered over a 10 s exposure and the low-loss region with an exposure of 2×10^−4^ s, both with a sum of three frames to generate a spectrum (Vigliaturo et al., 2019). The Fe-valence state was determined using the Δ*E* method described in Tan et al. (2012), with the same standards and experimental conditions as in Rojac et al. (2017). The use of the *L*_2,3_ white-line intensity-ratio method for determining the Fe-valence state is not the best solution for our sample, because the presence of Al^3+^ modifies the energy loss near edge structure (ELNES) of the Fe–*L*_2,3_ edge, and thus the calculated Fe^3+^ content is overestimated (Frost and Langenhorst, 2002; Langenhorst et al., 2013).

### 3.10 Morphometry by operator

The dimensional and morphological studies were conducted by OM (10×), FEG-ESEM (200×), FEG-ESEM (2000×), and STEM (25 000×) under the same conditions as those described above. The descriptive statistics are based on particle length (*L*), width (*w*), and aspect ratio (*L/w*), as well as additional parameters, such as the aerodynamic equivalent diameter (*D*_ae_), using the simplified formulation proposed by Gonda and El Khalik (1985). The frequency of a specific mineral habit was investigated according to Zoltai (1981), and Veblen and Wylie (1993). All visible particles were measured and assigned to specific categories, namely particles with *L/w* ≥ 3 (Gunter, 2018), EMPs (according to NIOSH, 2011), and fibers with *L/w* ≥ 3, *L* ≥ 5 μm, and *w* ≤ 3 μm (according to Belluso et al., 2017). We further classify the studied particles with respect to their potential to enter various parts of the respiratory system, i.e., as inhalable (*D*_ae_ <100 μm), PM_10_ (*D*_ae_ <10 μm; equivalent to thoracic fraction), respirable (*D*_ae_ < 4 μm), or PM_2.5_ (*D*_ae_ <2.5 μm) (ISO, 1995; CEN, 1993; ICRP, 1994).

The 300 *D*_ae_ data points obtained by SEM at 200×, SEM at 2000×, and STEM at 25 000× were organized in descending order and then merged together to obtain a complete dimensional distribution of particle sizes across 3 orders of magnitude. When merged, the datasets show overlaps (supplementary merged dataset); they contain particles in a size range that was measured at two different magnifications, i.e., at 200× as well as at 2000×, or at 2000× as well as at 25 000×. In these two overlapping regions of the dataset, several particles occur with similar *D*_ae_ (particle “pairs”). The *D*_ae_ values of these latter particles were used to calculate the arithmetic average *D*_ae_ of each pair. The two original particles were then discarded and replaced by the “new” particle with the calculated average *D*_ae_, which was used in the final merged dataset (*n* = 216). In regard to the data for *L*, *w*, and *L/w* for these particle pairs in the overlapping region of the datasets, a precaution principle was applied: for each particle pair, the particle conforming to a higher number of dimensional constraints (i.e., *L/w* ≥ 3, *L* ≥ 5, and *w* ≤ 3) was counted as valid when defining the total number of particles falling in a certain category P1 (*L/w* ≥ 3), P2 (*L/w* ≥ 3 and *L*>5), and P3 (*L/w* ≥ 3, *L* ≥ 5 μm, and *w* ≤ 3 μm). The information for *L*, *w*, and *L/w* corresponding to each particle with a certain *D*_ae_ was preserved. In the case that both particles in the pair were conforming to the same number of dimensional constraints (or none), the particle measured with the most accurate instrument (and higher magnification) was used to determine the descriptive statistics reported in Table 4.

## 4 Results

### 4.1 OM

The hand specimen contains aggregates of prismatic glaucophane crystals ranging in color from dark grey to very dark blue (Fig. 2a and b). The luster is vitreous to pearly (Fig. 2, Appendix Fig. B1). Individual glaucophane crystals are pre-dominantly in a random orientation throughout the sample (Fig. 2a), but in some regions columnar crystals occur in parallel orientation (Fig. 2b). The crystals that were detached from the main rock piece are mostly acicular and approximately a millimeter in length but in some cases smaller and equant (Figs. 2c, B1).

### 4.2 PXRD

**Figure C1.**
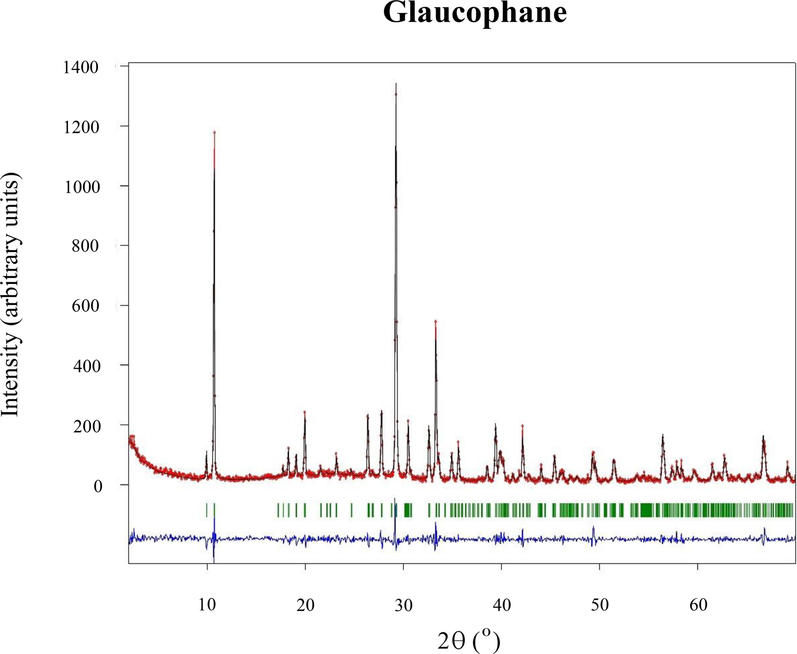
Observed (red points), calculated (black line), and difference (bottom blue line) X-ray powder diffraction pattern for the studied glaucophane sample. Bragg peak positions are given as vertical green bars.

The powder X-ray pattern (Fig. C1) for this sample perfectly matches the COD (Crystallographic Open Database) card 96–900-4136 (glaucophane). All observed Bragg peaks could be indexed in the monoclinic *C*2*/m* symmetry. The refinement of the cell parameters, achieved by using the FULLPROF Rietveld program (Rodríguez-Carvajal, 2001), yielded (Å) *a* = 9.5672(7), *b* = 17.7654(16), *c* = 5.3009(5), and *β*(°) = 103.717(6).

### 4.3 FTIR spectroscopy

**Figure 3.**
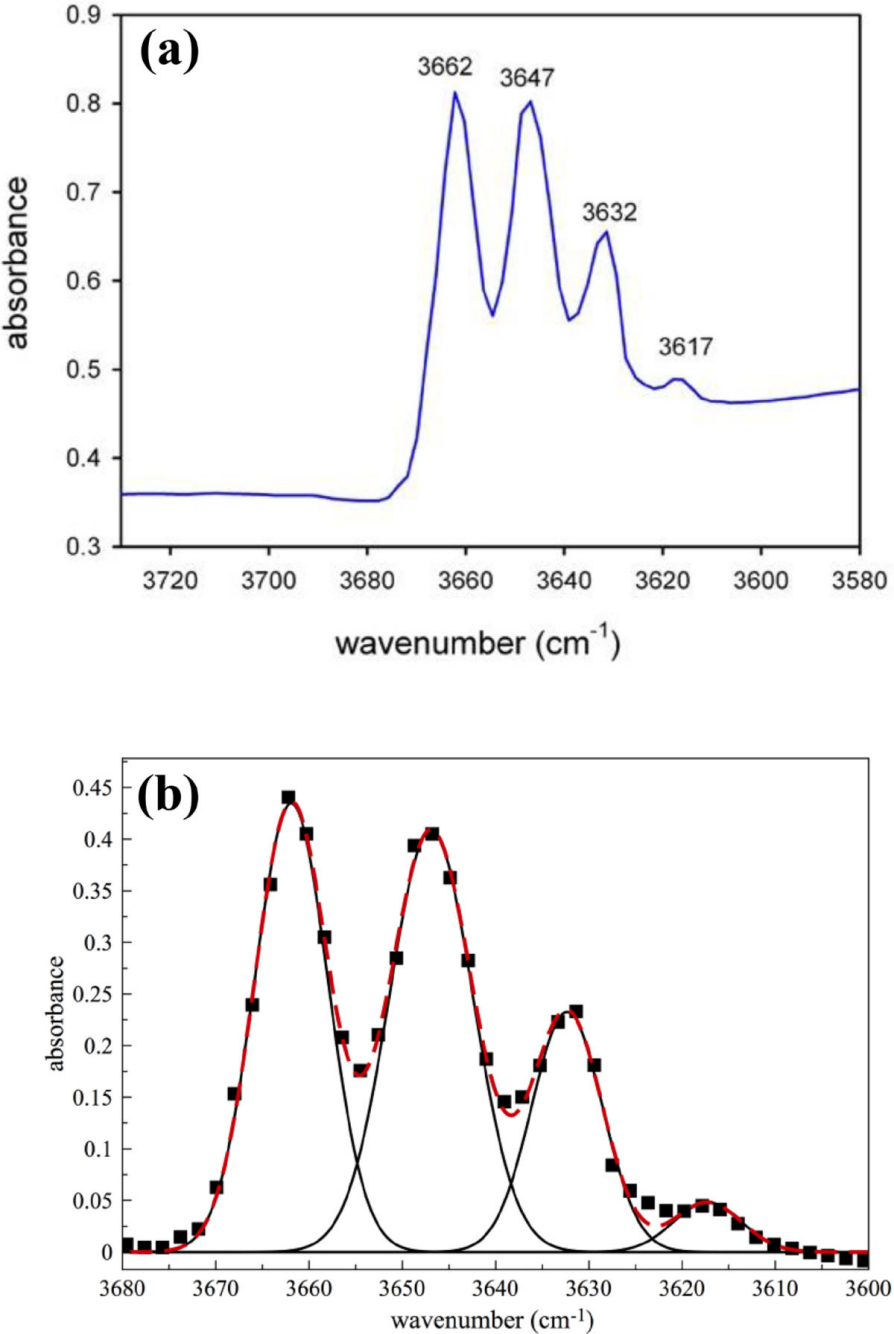
**(a)** Powder FTIR spectrum of the studied glaucophane in the OH-stretching region; **(b)** fitted spectrum. Squares: experimental pattern; black lines: fitted components; dashed red line: calculated spectrum.

The powder FTIR spectrum collected in the OH-stretching region (Fig. 3) shows the typical four-band pattern due to the distribution of Mg and Fe^2+^ at *M*(1,3). From the relative band areas of the fitted Gaussian components, and by using the method discussed in Iezzi et al. (2005), Della Ventura et al. (2016), and Della Ventura (2017), we calculated Mg at *M*(1,3) = 2.11 atoms per formula unit (a.p.f.u.), in agreement with the values provided by both electron microprobe analyzer (EMPA) and Mössbauer spectroscopy.

**Figure 4.**
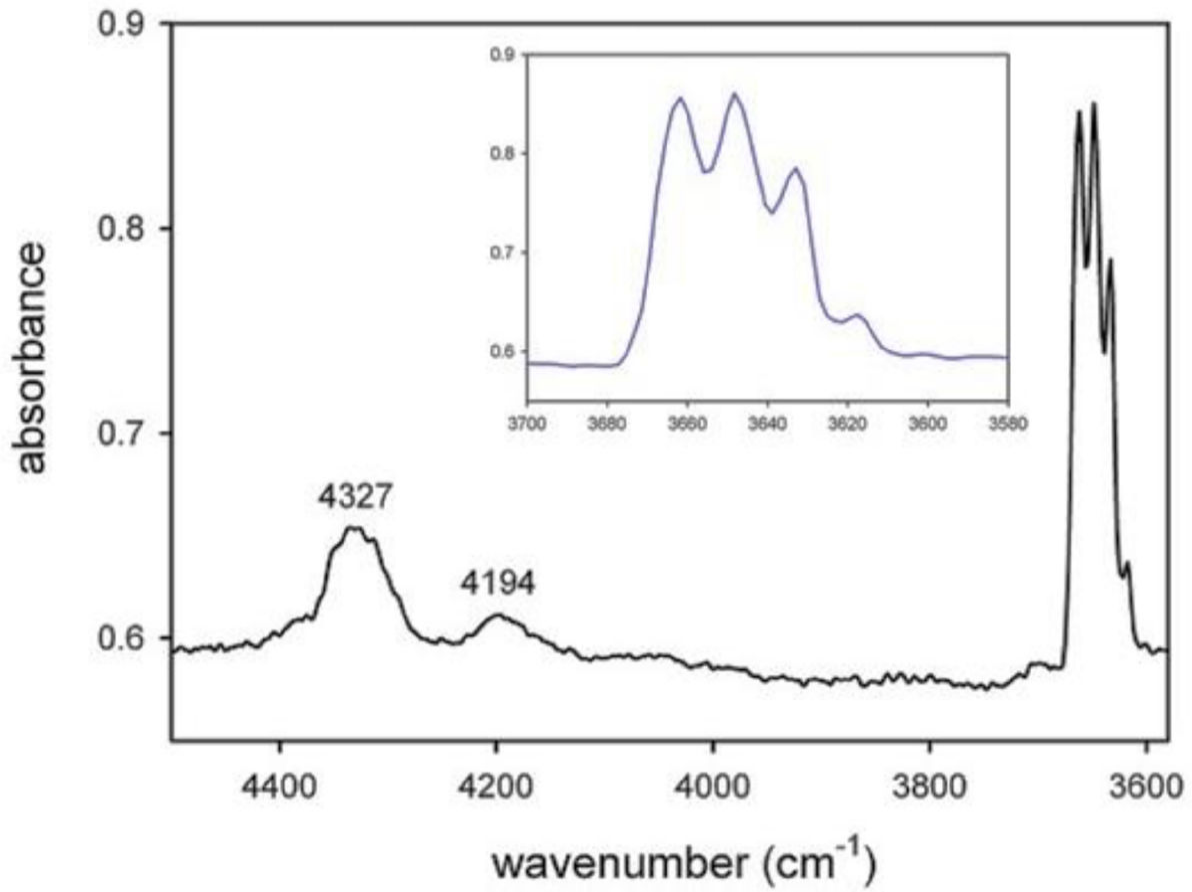
Single-crystal NIR spectrum of the studied glaucophane. The OH-stretching region is magnified in the inset.

The single-crystal NIR (near-infrared) spectrum (Fig. 4) shows, in addition to the quadruplet of peaks observed in the powder pattern (displayed as the inset in Fig. 4), two broad absorptions at 4327 and 4194 cm^−1^, which result from the combination of the stretching and libration vibrations of the O–H dipole (Della Ventura et al., 2021a).

**Figure 5.**
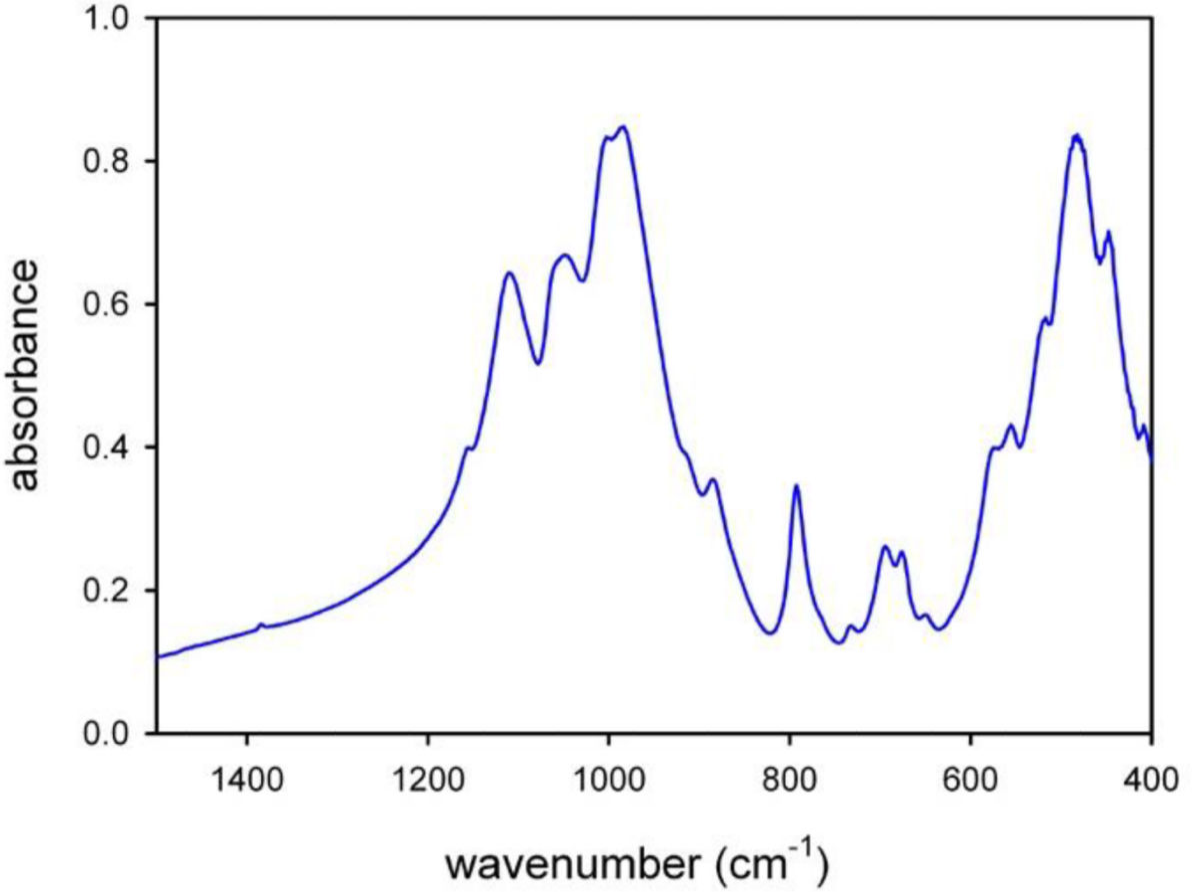
Powder FTIR spectrum of the studied glaucophane in the low-frequency lattice-mode region.

The powder FTIR spectrum in the low-frequency lattice region, between 1200 and 400 cm^−1^ (middle-IR or MIR), is relatively complex and shows several overlapping and broad peaks (Fig. 5). According to the work of Ishida et al. (2008) and Della Ventura et al. (2018, 2021a, b), the lattice-mode range of amphiboles may be conveniently divided into three regions: (1) the 1200–800 cm^−1^ range, where seven to eight very intense to medium intense bands due to lattice Si–O, Si–O–Si, and O–Si–O antisymmetric stretching vibrations are observed; in particular, the highest wavenumber peaks in this range can be assigned to *T* (1)–O(1), which is a very short bond in the amphibole structure (Della Ventura et al., 2021b); (2) The 800–600 cm^−1^ range, where four to six medium weak to very weak peaks resulting from Si–O–Si symmetric stretching or chain deformation modes occur; in this range, two O–H librations at around 700 and 690–640 cm^−1^ have been identified in ^T^Al-free and A-site empty amphiboles (Ishida et al., 2008), whereas the same modes are shifted to lower wavenumbers in A-site filled amphiboles (Della Ventura et al., 2021a); and (3) the 600–400 cm^−1^ range, where essentially Si–O bending vibrations and *M*–O modes are observed. The MIR spectrum of the glaucophane sample studied here is similar to the pattern given by Ishida (1990) for sodium amphiboles along the glaucophane–riebeckite series, in particular in the 1200–800 cm^−1^ range, where the patterns are almost identical. Small differences in the relative peak intensities and widths are observed in the 800–400 cm^−1^ range, where the effect of cationic substitutions in these systems is significant (Ishida, 1990).

### 4.4 μ-Raman spectroscopy

**Figure 6.**
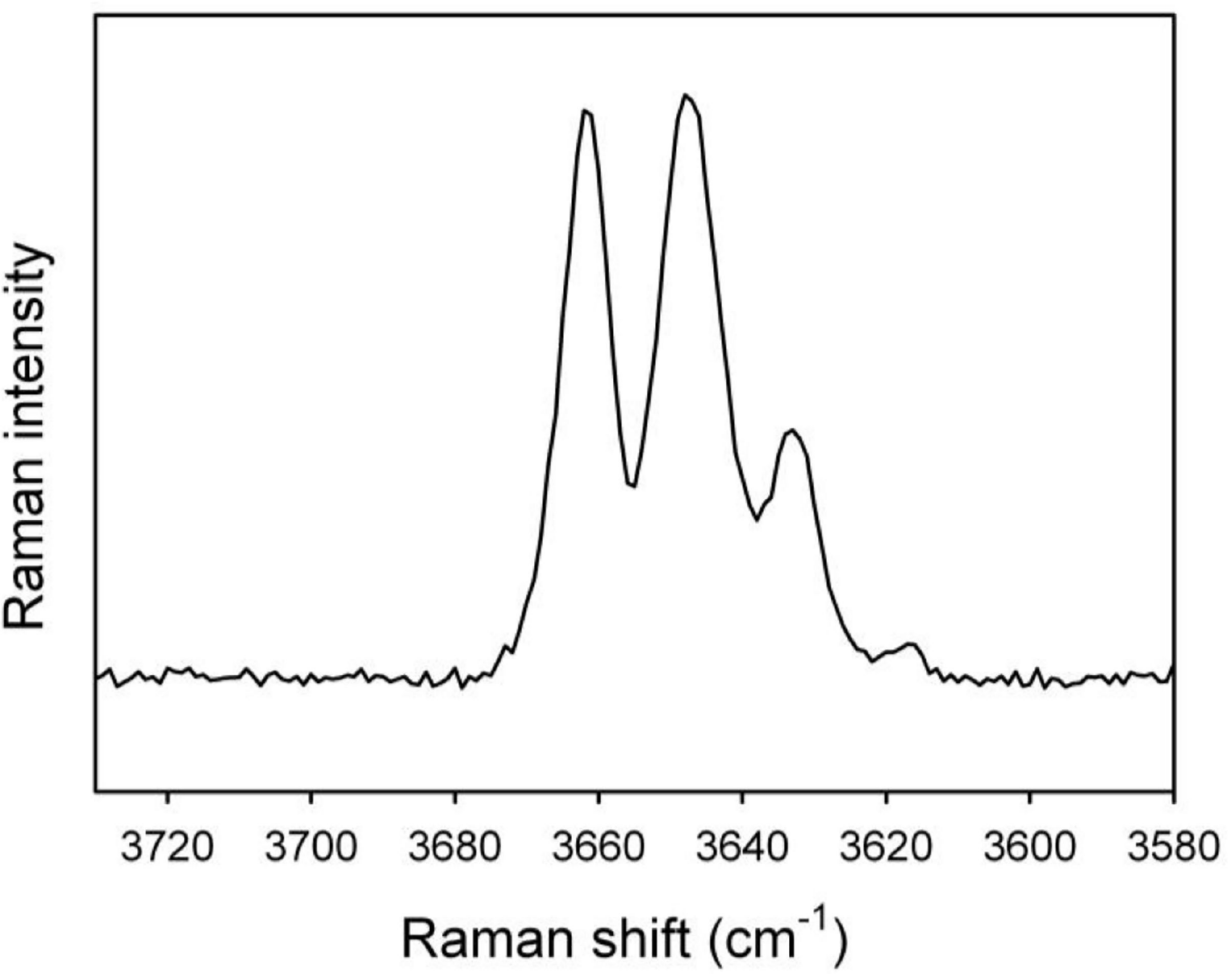
Raman spectrum of the studied glaucophane in the OH-stretching region.

As expected (e.g., Leissner et al., 2015; Della Ventura et al., 2021a, b), the OH-stretching region of the Raman spectrum obtained for the studied glaucophane (Fig. 6) is almost identical to the OH-stretching FTIR pattern (Fig. 3) and shows the same quadruplet of peaks having the same relative intensities as those observed by fitting the IR curve. This important observation implies that both techniques provide the same crystal–chemical results.

**Figure 7.**
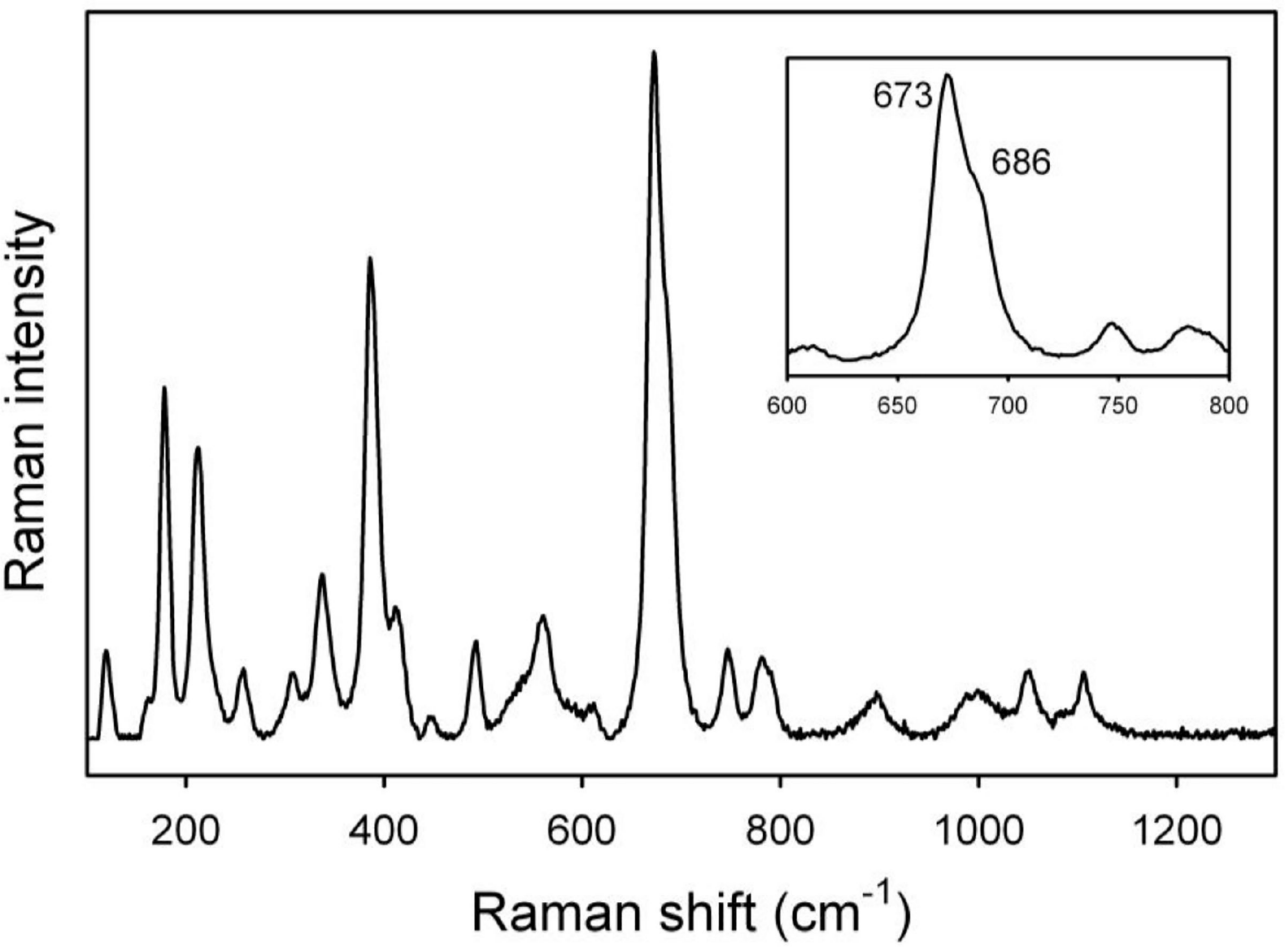
Raman spectrum of the studied glaucophane in the lattice-mode region. The inset displays a magnification of the ring-breathing mode range at 650–700 cm^−1^.

The Raman spectrum in the lattice-mode region is displayed in Fig. 7. Similar to the FTIR spectra, the Raman spectra of amphiboles (e.g., Leissner et al., 2015; Waeselmann et al., 2019) can be conveniently divided into three main regions: (1) between 850 and 1200 cm^−1^, where medium- to low-intensity peaks due to *T* –O stretching mode occur; (2) between 550 and 850 cm^−1^, where the strongest Raman peak occurs for the majority of amphiboles, with the notable exception of riebeckite (Susta et al., 2018). This peak, which occurs near 670±20 cm^−1^ (Waeselmann et al., 2019), is assigned to deformation of the tetrahedral double chain, and its position may provide a useful tool for amphibole identification (e.g., Della Ventura, 2017, and references therein). As recently shown by Bersani et al. (2019) and Della Ventura et al. (2021b), this peak linearly shifts in position as a function of the Mg–*Me*^2+^ (*Me* is the divalent metal cation) substitution at the octahedral sites; and (3) the range between 100 and 550 cm^−1^, which is complicated by the presence of many medium- to low-intensity peaks that are strongly polarized (Waeselmann et al., 2019). These peaks have been difficult to assign with confidence because of the overlapping of *M*–O modes, *T* –O bendings, and O–H librations (Della Ventura et al., 2021a). According to Della Ventura et al. (2021b), most peaks in this range also shift linearly toward a lower wavenumber as a function of octahedral substitutions.

Overall, the pattern in Fig. 7 is almost identical to that given for glaucophane in Waeselmann et al. (2019, sample 22). In particular, a well-resolved component on the higherfrequency side of the ring-breathing mode at 673 cm^−1^ (see inset in Fig. 7) is consistent with the presence of ^C^Al in the amphibole, whereas the peak at 530–570 cm^−1^ can be assigned to octahedral Fe^3+^; the low intensity of these peaks is in agreement with the low amount of Fe^3+^ indicated by Mössbauer spectroscopy.

### 4.5 Mössbauer spectroscopy

**Figure 8.**
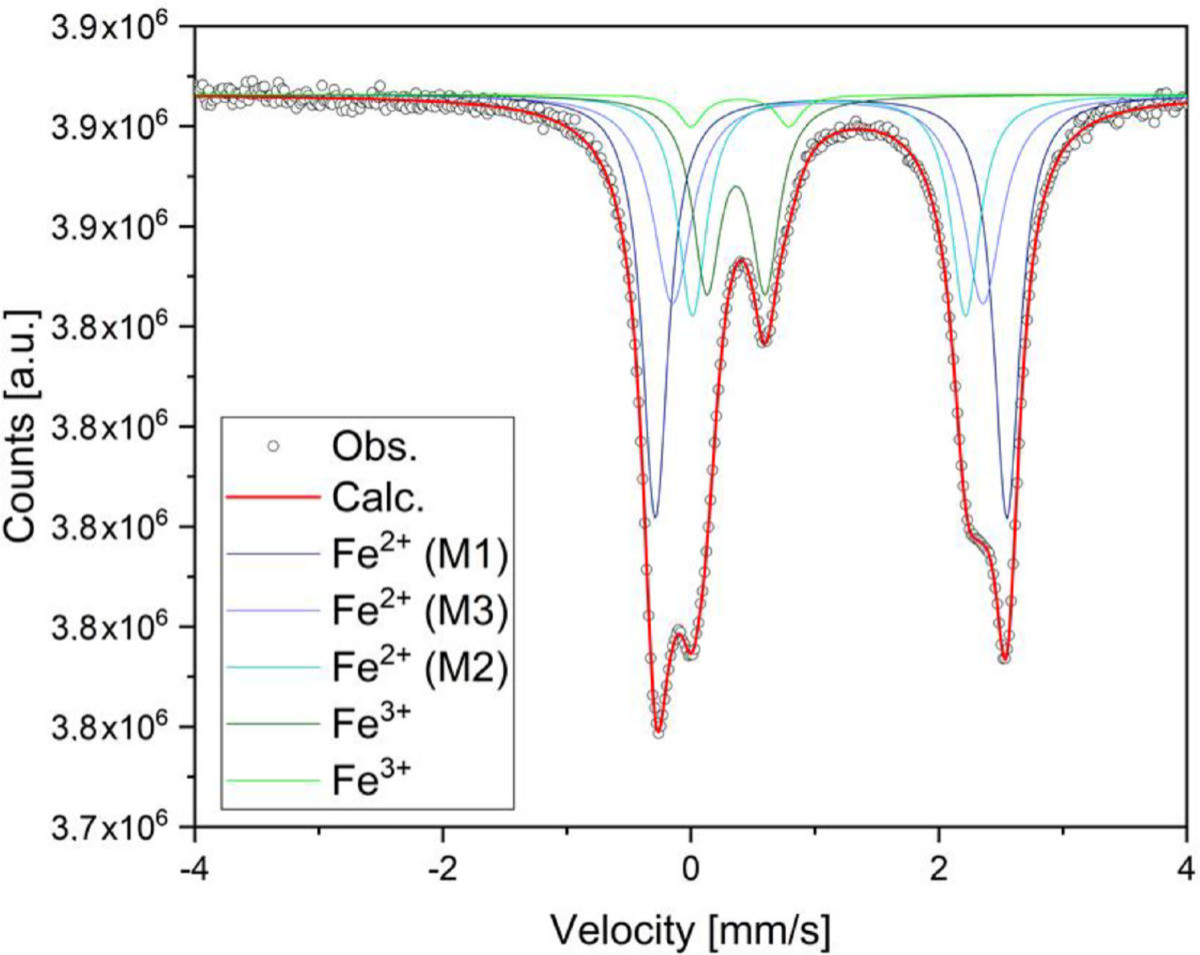
Fitted ^57^Fe Mössbauer spectrum for glaucophane.

The ^57^Fe Mössbauer spectrum (Fig. 8) shows well-separated absorption lines with a distinct shoulder at ~+2.5 mm/s. Satisfactory evaluation of the data is only possible when the three typical doublets due to Fe^2+^ at *M*(1), *M*(2), and *M*(3) and one Fe^3+^ component are used, all assigned to Fe in the octahedral strip, based on the ^57^Fe hyperfine parameters. A slight asymmetry and small misfits around +0.8 mm/s in these four-component fits were accounted for by introducing a second, low-intensity Fe^3+^ doublet. This additional doublet significantly improved the refinement and led to stable fits. No clear evidence was found for Fe^2+^ at the *M*(4) site. The individual Fe^2+^ components were assigned in accordance with previous studies of amphiboles (e.g., Redhammer and Roth, 2002; Iezzi et al., 2005; Della Ventura et al., 2005, 2016), and the result is given in Table 1, together with the extracted relative area fractions. The refined Fe^3+^ content is ~20% of the total Fe, whereas ~60% of the total Fe is allotted to the OH-coordinated *M*(1,3) sites, with the remainder being assigned to the *M*(2) octahedron.

### 4.6 EPMA

The EPMA data shown in Table 2 represent the average of 35 analytical points on two crystals, with the FeO and Fe_2_O_3_ contents based on the Mössbauer results. The crystal–chemical formula has been calculated on the basis of 23 oxygen atoms using the spreadsheet of Locock (2014). Ferrous and ferric iron were assigned to the various sites based on the Mössbauer data of Table 1, and the final site occupancies are given in Table 3.

### 4.7 FEG-ESEM EDXS

**Figure 9.**
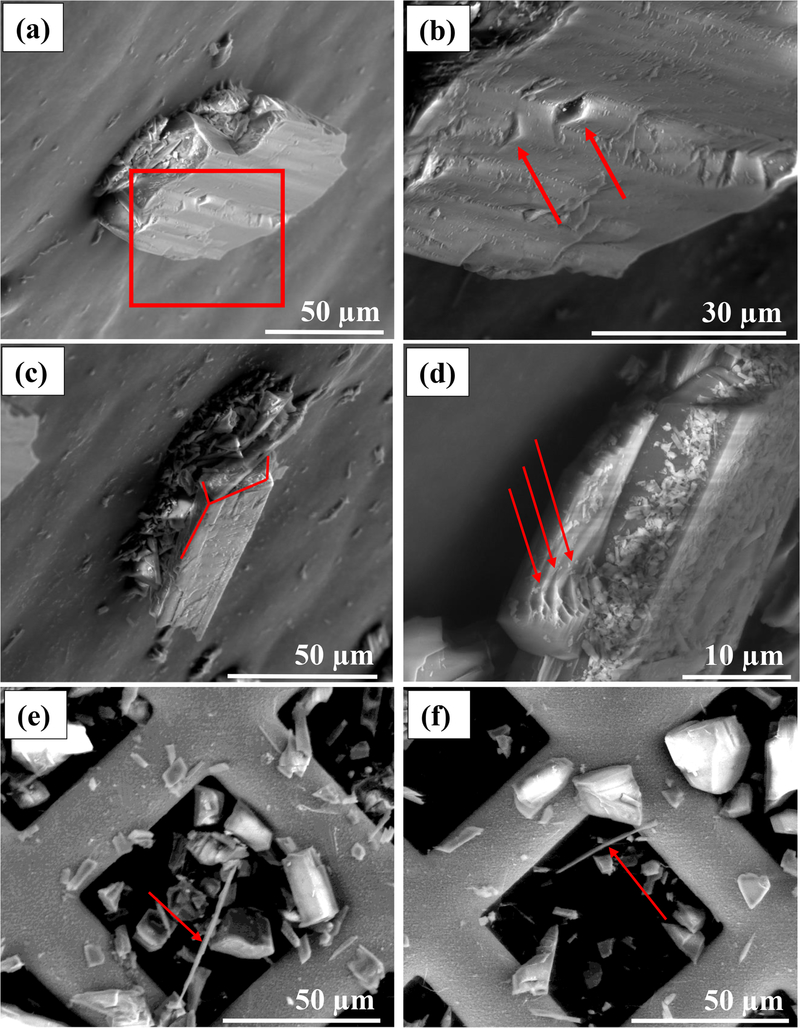
Glaucophane particle morphology observed on a 70° inclined SEM stub. **(a)** Stepped glaucophane particle. The red square represents a region of interest (ROI) shown in greater detail in panel **(b)**. **(b)** Stepped structure with slightly rounded edges. **(c)** An elongate prismatic particle in which the characteristic amphibole angles are visible (56° and 124°). **(d)** Lamellar structures (red arrows) with rounded and sharp edges. **(e, f)** SEM picture of a TEM grid showing the presence of large fibrous particles (red arrows).

The habit of the studied particles is mostly prismatic and acicular. Surfaces parallel to the (002) plane occasionally show steps and irregularities (Fig. 9a and b). The characteristic angles between cleavage planes (56 and 124°) can be observed in some of the crystals (Fig. 9c). A large number of particles shows the presence of lamellar structures and possible evidence of dissolution at the edges (Fig. 9d). TEM grids investigated by FEG-ESEM show the presence of a diverse set of habits and particle morphologies (Fig. 9e and f). Some rare fibrous particles were visible (red arrows), having a habit that might be recognized as asbestiform or as fiber (Fig. 9e and f).

### 4.8 HR-TEM-SAED and acSTEM Dual-EELS

**Figure 10.**
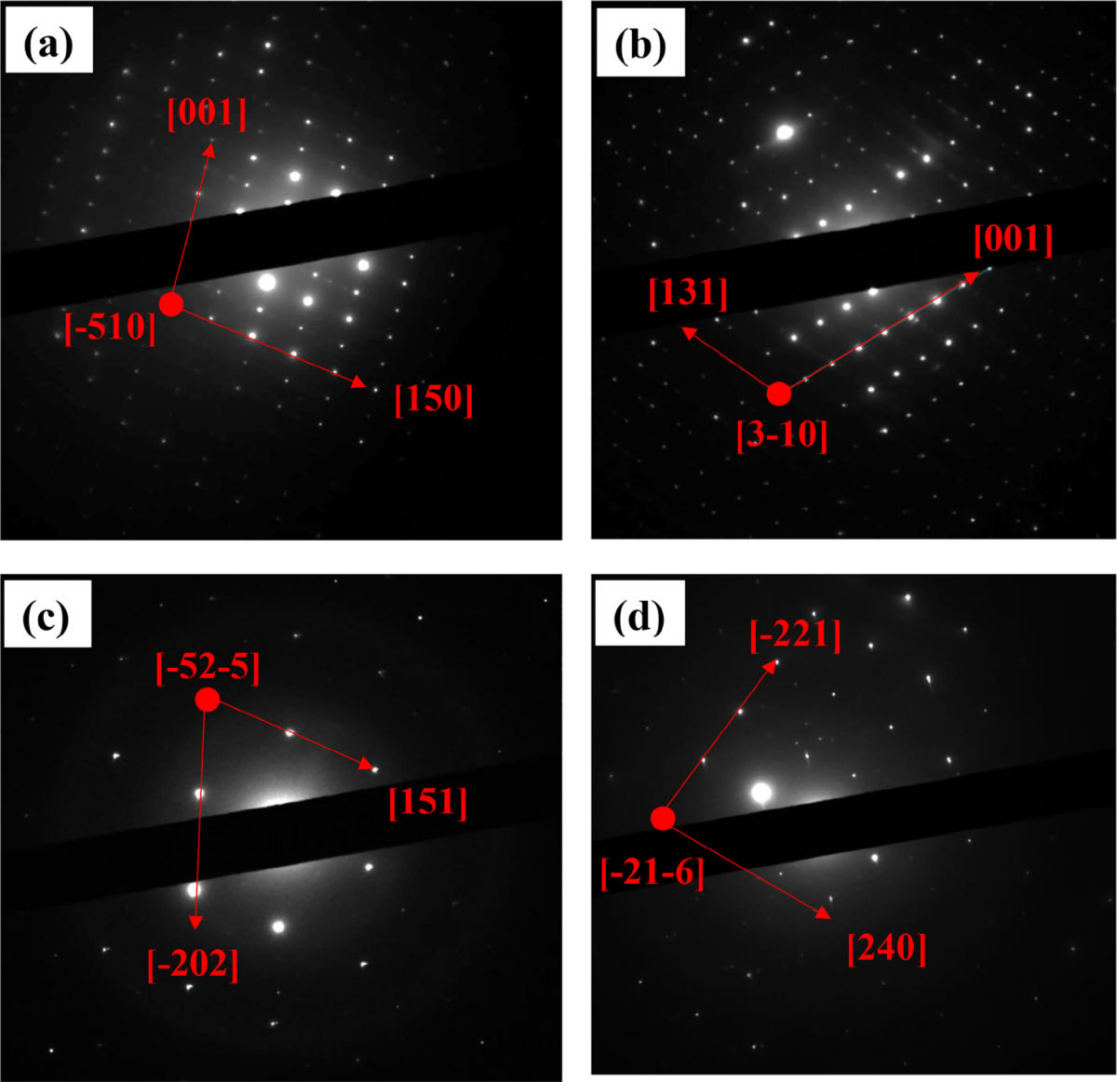
**(a–d)** SAED patterns collected using the JEOL 2010 TEM on different glaucophane crystals showing different orientations, crystallographic directions (red arrows), and lattice defects (streaking in panels **a** and **b)**. The zone axes are indicated by the red dots.

**Figure 11.**
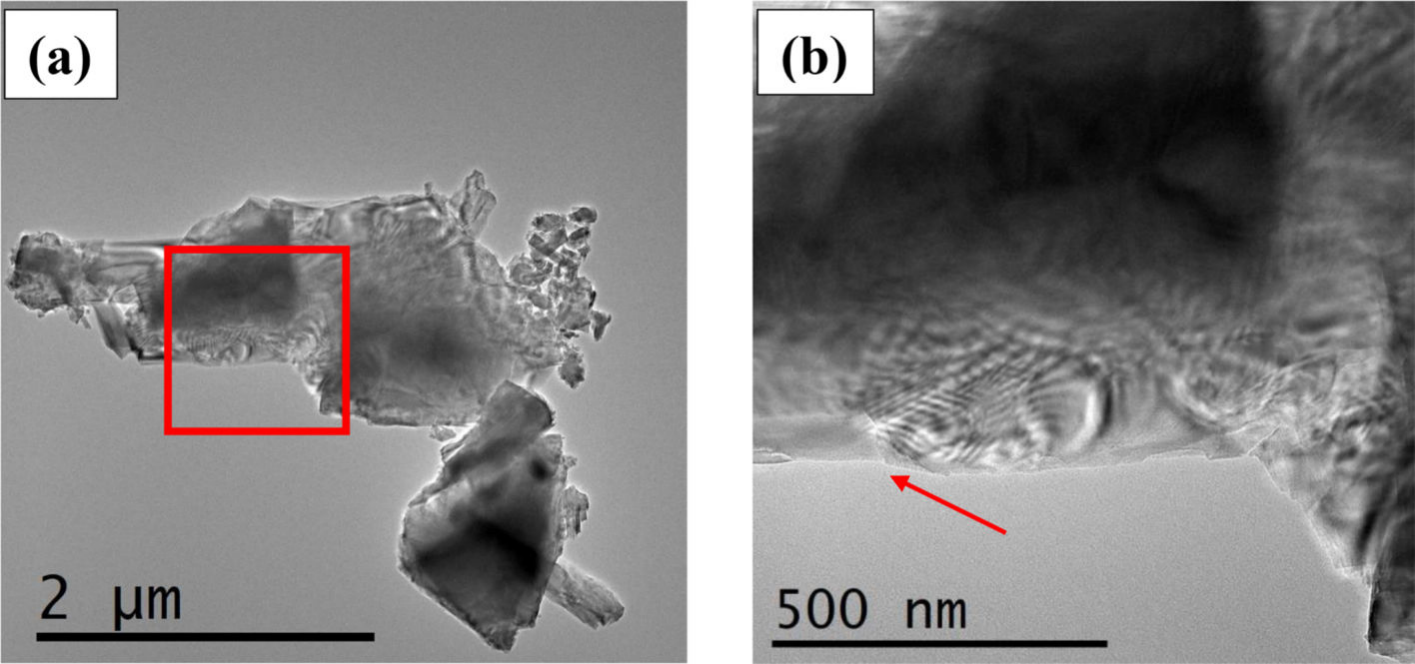
**(a)** Bright-field (BF) TEM image showing diffraction fringes on a glaucophane particle. The red square represents the area shown in panel **(b)**. **(b)** Magnified image of the area with diffraction contrast that is generated by the presence by slightly misoriented lamellae. A step in the grain-boundary area is visible (red arrow).

The investigated mineral particles show in some cases defects, as evidenced by the weak streaking visible along the [150] and the [131] directions, and cutting the [001] direction in the SAED patterns (Fig. 10). The stepped lamellar structures observed in the FEG-ESEM (Fig. 9D) are also visible at the TEM scale in regions where diffraction contrasts appear (Fig. 11). The misorientation of the laminae may generate the presence of diffraction contrast and possibly Moirée fringes (Fig. 11b).

**Figure 12.**
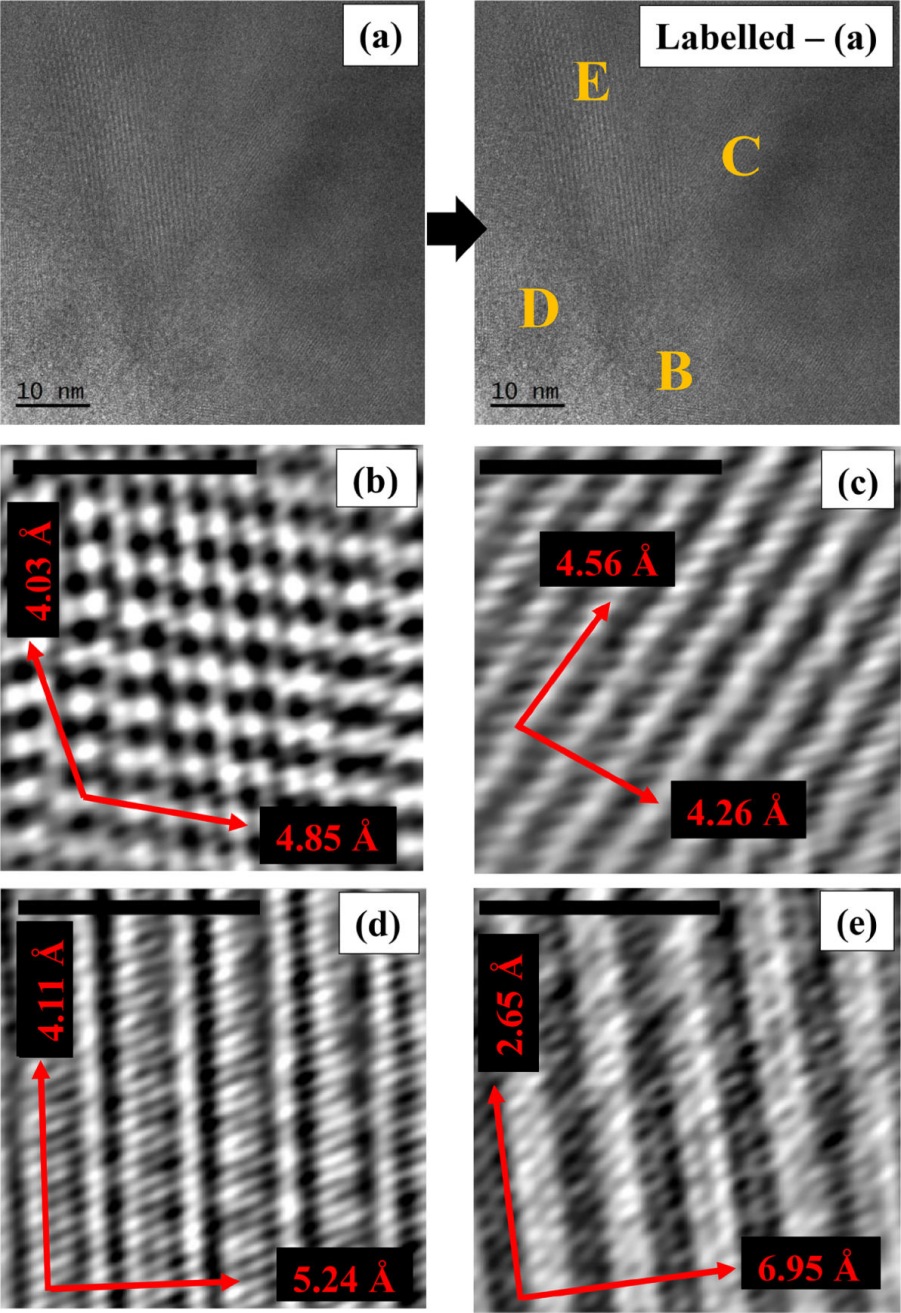
**(a)** High-resolution TEM micrograph of a region in which several orientations of the lattice are visible. The yellow labels (B, C, D, E) show the ROIs used to determine the main crystallographic directions and the *d* spacings of the corresponding planes displayed at higher magnifications in the IFFT images shown in panels **(b)**, **(c)**, **(d)**, and **(e)**. Standard deviations (*σ*_*n*−1_) for the *d* spacings of the planes that correspond to the crystallographic directions shown by red arrows are indicated in the text only. Scale bars in panels **(b)**, **(c)**, **(d)**, and **(e)** are 2 nm.

**Figure 13.**
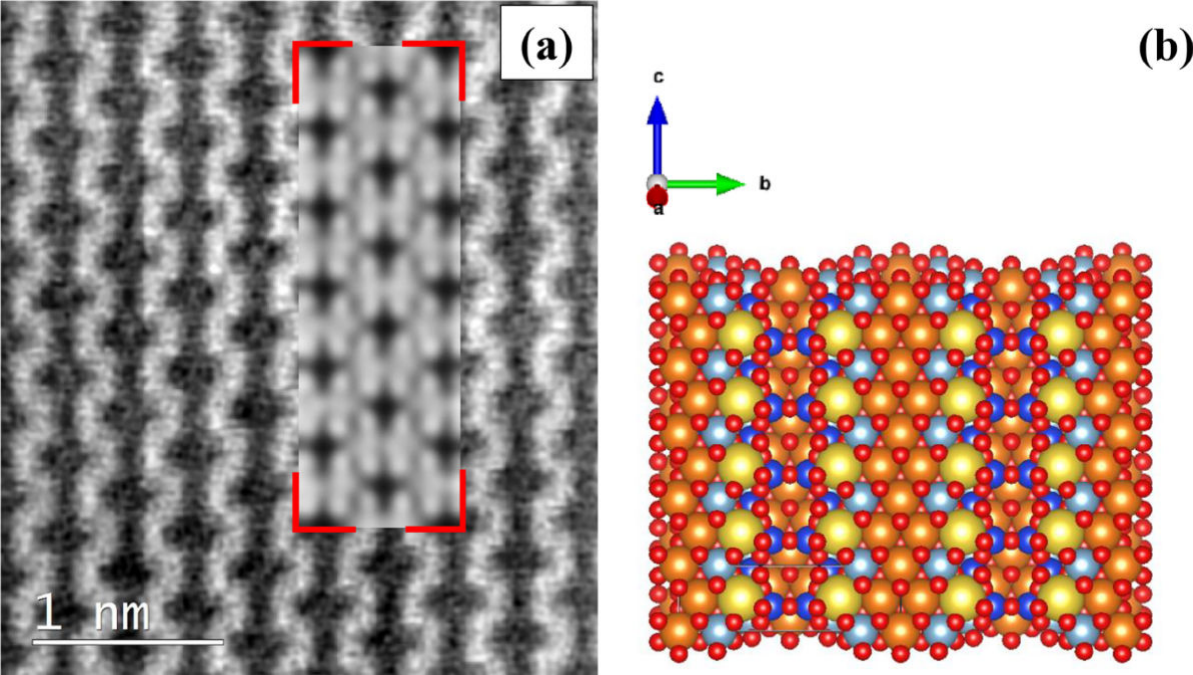
**(a)** Denoised atomic-resolution acSTEM picture of the chain-like structure of glaucophane. The simulated structure (delimited by red corners) is superimposed onto the real image. **(b)** Structural model, oriented to be consistent with the structure imaged in panel **(a)**, was used to generate the simulated atomic-resolved crystal structure (delimited by red corners) in panel **(a)**. Si atoms are blue, Mg- or Fe-atom positions are orange, O atoms are red, Al atoms are light blue, and Na atoms are yellow.

In several areas of the studied particles, HR-TEM images reveal the presence of multiple orientations (Fig. 12). All the *d* spacings of crystallographic planes and the associated direction angles were determined on the fast Fourier transform (FFT) image (not shown) that corresponded to the inverse fast Fourier transform (IFFT) image (Fig. 12) of a selected region (B, C, D, and E). In Region B, the *d* spacings of 4.03Å (*σ*_*n*−1_ = 0.23) and 4.85Å (*σ*_*n*−1_ = 0.33), measured along the crystallographic directions (red arrows), may be assigned to the (111) and (−111) planes, respectively. The two directions are separated by an angle of ~120.65°. In Region C, *d* spacings of 4.56Å (*σ*_*n*−1_ = 0.08) and 4.26Å (*σ*_*n*−1_ = 0.07) were measured along the crystallographic directions shown by red arrows and may be assigned to the (200) and (020) planes, respectively. The two crystallographic directions are separated by an angle of ~82.83°. In Region D, the two *d* spacings measured along the crystallographic directions (red arrows) are 4.11Å (*σ*_*n*−1_ = 0.08), which may be assigned to the (−131) plane, and 5.24Å (*σ*_*n*−1_ = 0.12), which may be assigned to the plane perpendicular to the *c* direction (compatible with the unit-cell dimension). The angle between the two identified crystallographic directions is ~93.70°. In Region E, two crystallographic directions (shown by red arrows) are seen, which correspond to planes with the *d* spacings of 2.65Å (*σ*_*n*−1_ = 0.07) and 6.95Å (*σ*_*n*−1_ = 0.14), measured along these directions. The first spacing may be assigned to the (002) planes, whereas the second cannot be assigned to a specific direction. The two directions are separated by an angle of ~89.55°. The unassigned *d* spacing (6.95 Å), shown in Fig. 12e, was further investigated by atomic resolution microscopy, which allowed us to simulate the chain-like structure visible at atomic resolution (Fig. 13a) by using a glaucophane model oriented with the *c* and *b* axes perpendicular to the electron beam (Fig. 13b).

**Figure 14.**
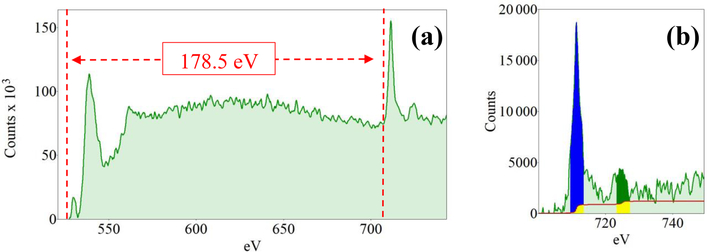
**(a)** Example of a core-loss spectrum showing both the O–*K* edge and Fe–*L*_2,3_ edges. **(b)** Zoom on the Fe–*L*_2,3_ edges used for the determination of the white-line intensity ratios. It displays the pre-edge background-subtracted core-loss region of a characteristic EELS spectrum with a focus on the Fe edges. The green area represents the collected original spectrum; the red line represents the double arctangent step function used to subtract the underlying background. The 4 eV wide integration windows are shown in blue on the *L*_3_ edge and in dark green for the *L*_2_ edge. The yellow region represents the area of the window that is subtracted by the double arctangent step function.

The average Fe-valence state at the particle grain boundary was estimated, using the Δ*E* method, at 2.55 (*σ*_*n*−1_ = 0.31; *n* = 10). Despite the well-known experimental limitation due to the Fe-edge modification by the presence of Al^3+^, we also applied the *L*_2,3_ white-line intensity-ratio method (Fig. 14), which returned a valence state of 2.78 (*σ*_*n*−1_ = 0.27; *n* = 10), a value that is statistically identical to that determined by the Δ*E* method.

**Figure 15.**
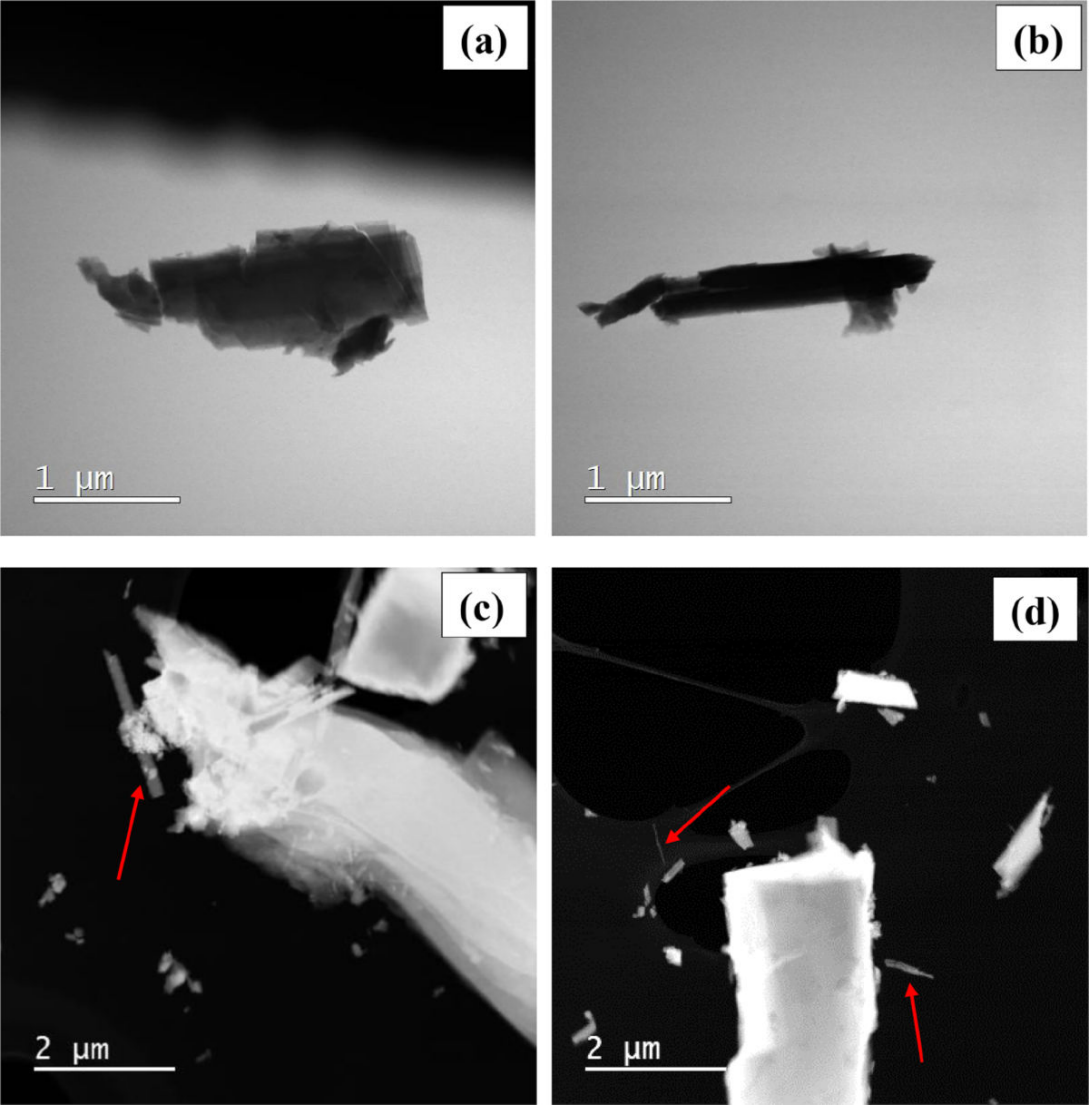
**(a)** STEM picture of the starting (0°) and **(b)** final position (120°) of the particle used for the three-dimensional tomographic reconstruction. **(c, d)** Dark-field STEM pictures showing the presence of nano-sized EMPs (red arrows).

The particle displayed in Fig. 15 was used for investigating its dimensional properties using 3-D TEM tomography (see supplementary video): at the starting position of the tomographic sequence, *L* was 1.86 μm and *w* was 0.81, and thus *L/w* = 2.30, whereas at the final position (after a rotation of 120°), *L* was 1.97 μm, *w* was 0.31 μm, and *L/w* was 6.36. Occasionally, nano-EMPs were observed in STEM mode (Fig. 15c and d).

### 4.9 Morphometry by operator

**Figure 16.**
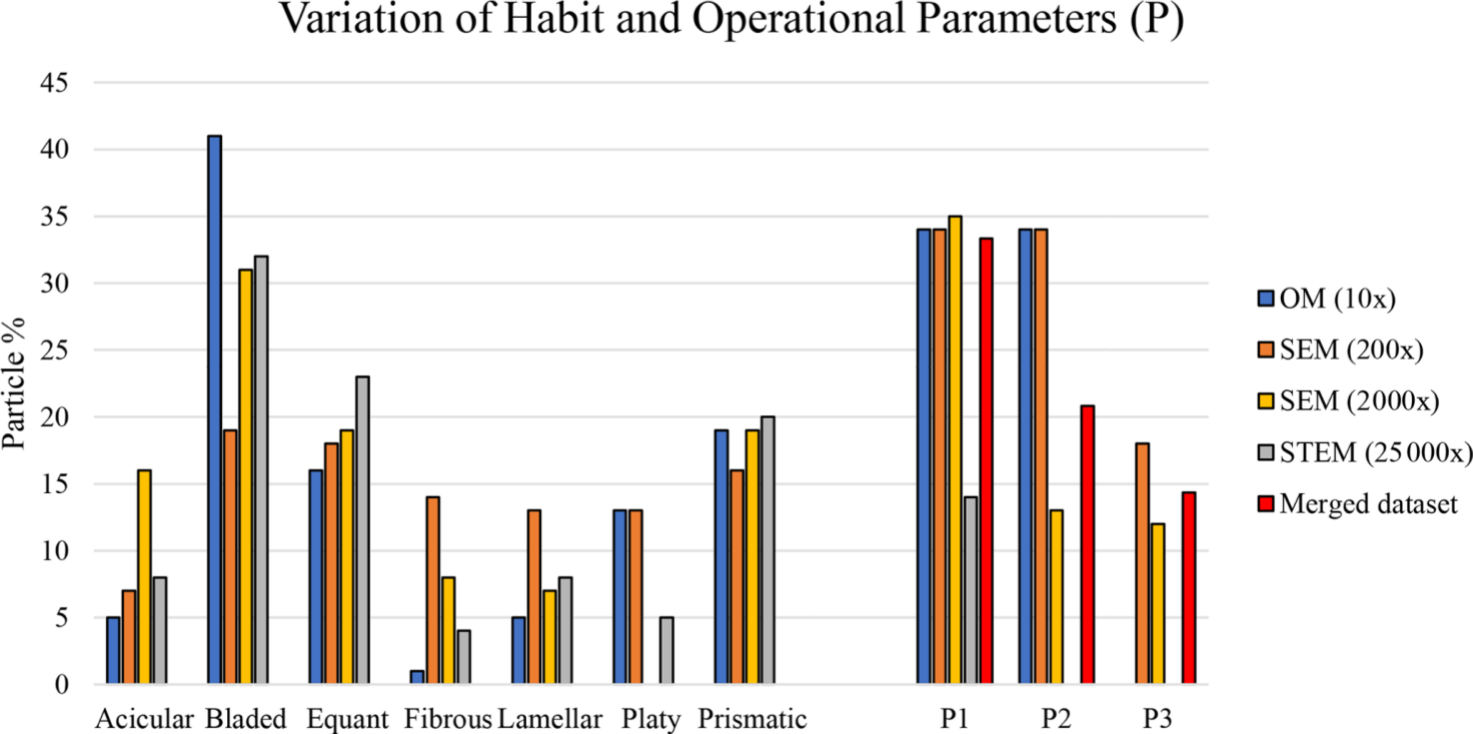
Habit variation using different types of microscopy and magnification. Habit nomenclature after Zoltai (1981), and Veblen and Wylie (1993). The variation of the different operational parameters is shown as P1, P2, and P3, where P1 represents *L/w* ≥ 3 only (Gunter, 2018; including the nano-sized portion of the mineral particle population), P2 shows *L/w* ≥ 3 and *L*>5 (i.e., EMP according to the National Institute for Occupational Safety and Health (NIOSH) definition), and P3 displays *L/w* ≥ 3, *L* ≥ 5 and *w* ≤ 3 (i.e., Belluso et al., 2017).

**Figure 17.**
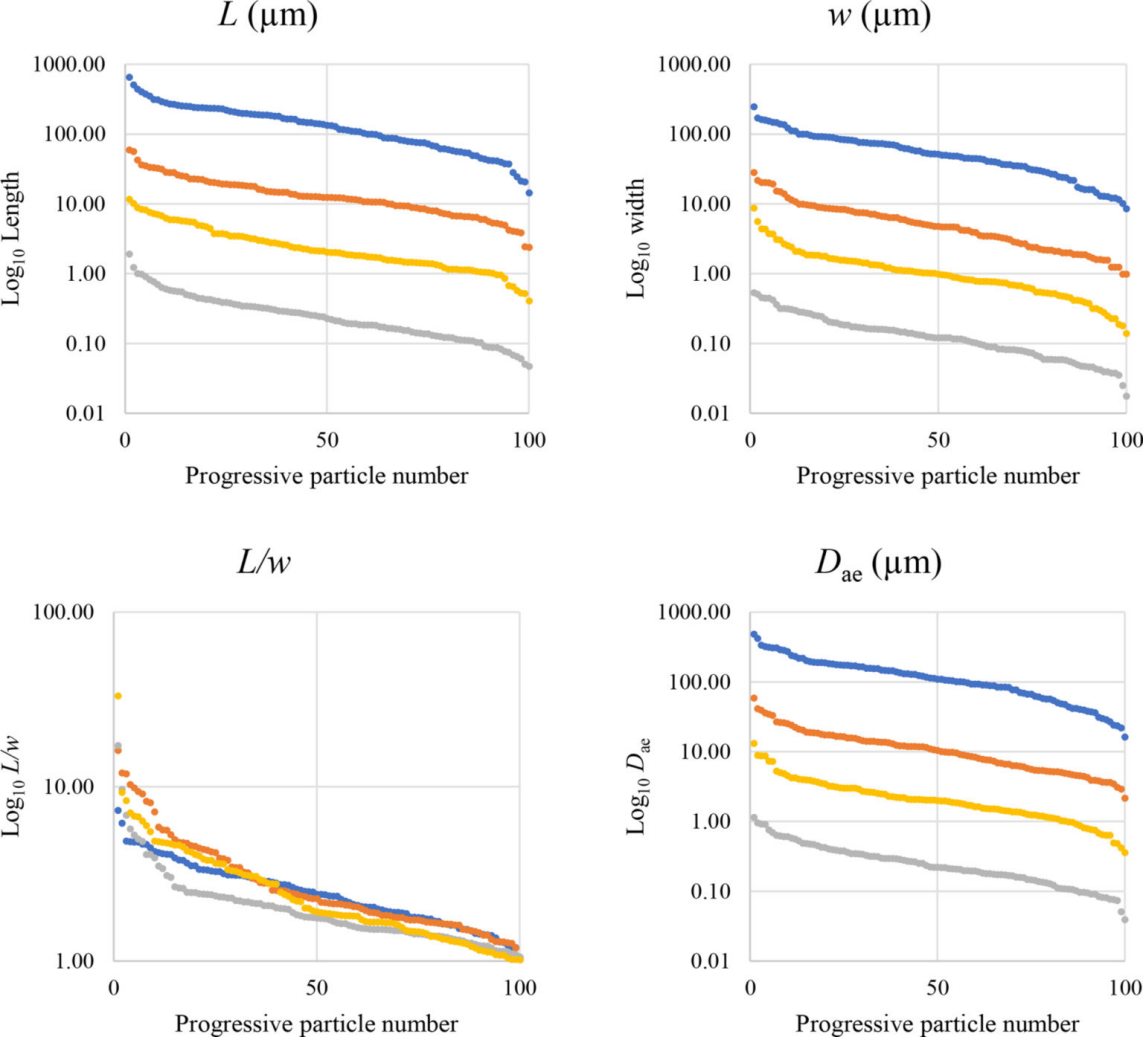
Plot of the dimensional data in descending order. Blue dots are particles measured by optical microscopy (10×), orange dots are particles measured with SEM (200×), yellow dots are particles measured with SEM (2000×), and grey dots represent particles measured by STEM (25 000×).

**Figure 18.**
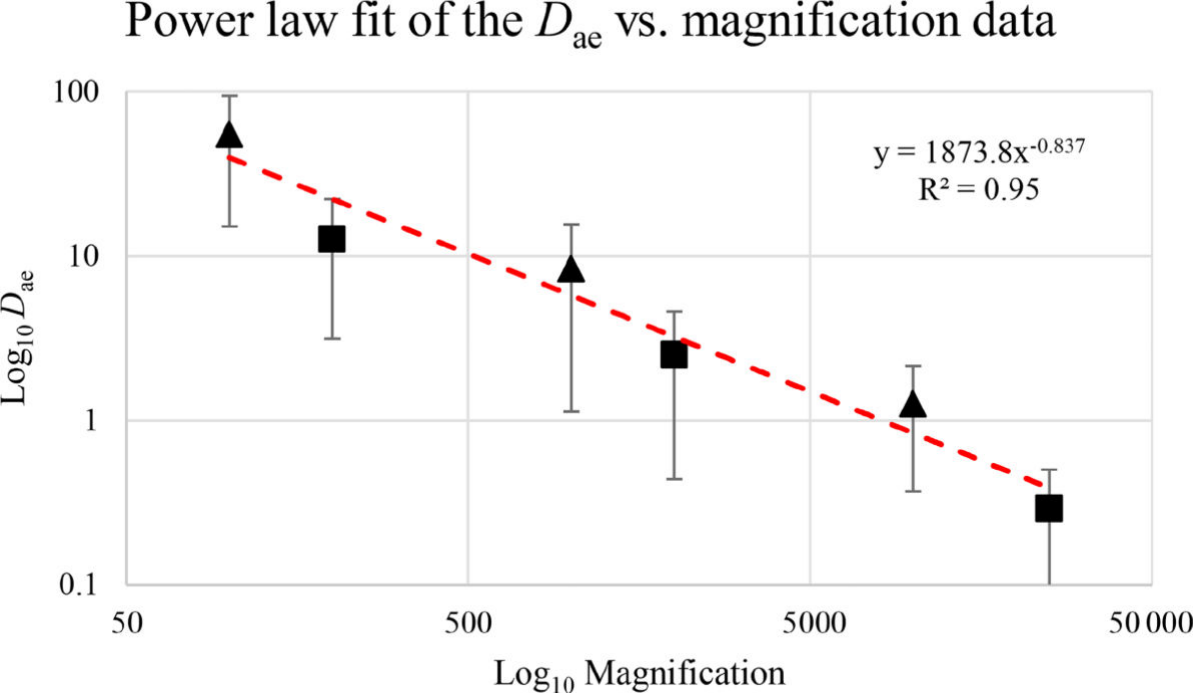
Average *D*ae at different magnifications for actinolite–tremolite (triangles; from Vigliaturo et al., 2018) and glaucophane (squares; this paper). The dashed red line shows the power-law fit.

Figure 16 reveals that the habit as well as the dimension and assigned category by operational parameters (P) of the investigated particles vary depending on the type of microscopy and the magnification used (Table 4). The nano-sized portion of particles (i.e., *D*_ae_ <100 nm) was detected by STEM but not by SEM (Table 4). We have also plotted the morphological parameters (*L*, *w*, *L/w*, and *D*_ae_) for all analyzed particles (*n* = 100 for each magnification) against progressive particle number, whereby the particle numbers have been arranged from the largest to the smallest dimensions (Fig. 17). The particle-size distribution, derived from the same datasets, and obtained by arbitrarily binning the dimensional parameters into 20 bins, is provided as histograms in Appendix D. The expected average *D*_ae_ for any selected magnification can be determined using the power-law relationship between the known average *D*_ae_ for each studied particle population and the corresponding magnification. To confirm this relationship, we used the EM data from other amphiboles (tremolite–actinolite), which were dimensionally investigated with the same SEM instrument and the same sample-preparation method (Vigliaturo et al., 2018). The plot of all available average *D*_ae_ values vs. magnification shows a robust fit (*R*^2^ = 0.95) of the power law (Fig. 18). From these data, we can estimate the expected average *D*_ae_ at any magnification.

## 5 Discussion

The amphibole in the studied sample was identified as glaucophane by the use of PXRD (Fig. C1). A combination of analytical techniques yielded a crystal chemical composition typical of glaucophane, with limited Fe^2+^ substituting for Mg in the *M*(1,3) octahedra and limited Fe^3+^ and Fe^2+^ replacing Al at the *M*(2) site.

The sample is highly crystalline and contains some defects, which appear as streaking that is occasionally visible along the [150] and the [131] crystallographic directions, and intersecting with the [001] crystallographic direction. The streaking might originate from Wadsley defects in the amphibole crystal structure (Chisholm, 1973). All measured *d* spacings can be assigned to specific crystal planes (and corresponding crystallographic directions), except for one spacing (6.95 Å, *σ*_*n*−1_ = 0.13) along the direction (red arrow on lower right) shown in Fig. 12e. The investigation at atomic resolution and the related simulated image, however, show that this unassigned spacing could be generated by the succession of double chains perpendicular to the [020] crystallographic direction (Fig. 13). Since we measured this unassigned *d* spacing on different instruments, and the simulated image fits the real atomic-resolution image of the crystal structure, we do not think that this is an artifact but rather that it might be generated by a “stepped” superimposition of I beams and the occasional presence of Wadsley defects in the crystal structure.

The Fe-valence state determined by EELS in the grain-boundary regions of the particles is higher when using the *L*_2,3_ method (2.78; *σ*_*n*−1_ = 0.27; *n* = 10) than when using the Δ*E* method (2.55; *σ*_*n*−1_ = 0.31; *n* = 10). This difference in the Fe-valence state is a consequence of the presence of Al^3+^, which modifies the *L*_2_ and *L*_3_ peaks in the EELS spectrum and thus the shape of the *L*_2,3_–Fe edge (Frost and Langenhorst, 2002; Langenhorst et al., 2013). Therefore, whenevaluating the valence state of Al-containing amphiboles, the Δ*E* method is the better option. The value determined by the Δ*E* method for the grain-boundary region of the glaucophane (2.55) is higher than the bulk Fe-valence state obtained by Mössbauer spectroscopy (~2.20). This discrepancy is a consequence of (i) oxidation of the most external boundary of the glaucophane crystals, and (ii) the possible accumulation of Fe oxyhydroxides at the particle surfaces (Fantauzzi et al., 2010; Vigliaturo et al., 2019).

The studied sample shows a predominance of randomly oriented (Fig. 2a) crystals when observed at the mesoscale. The dark to very dark blue color (Fig. 2a and b) and the vitreous to pearly luster (Fig. 2 and Appendix B) are compatible with glaucophane. Some regions of the sample are texturally more ordered, with parallel crystals having a columnarlike habit (Fig. 2b). The millimetric crystals, which were easily detached from the main rock piece, mainly showed an acicular habit and less frequently smaller equant particles (Fig. 2c). At this level of investigation, we did not observe any asbestiform morphology, because we did not detect (a) small fiber thickness and large fiber length, (b) flexible and easily separable fibers, and (c) a parallel arrangement of the fiber in the unprocessed sample (Zoltai, 1981; Veblen and Wylie, 1993). The FEG-ESEM images of glaucophane particles confirmed the predominance of prismatic and acicular fragments (Fig. 9). Of note is that we observed a high abundance of lamellar structures (Fig. 9d), which were also detected with the TEM (e.g., Fig. 11). Interestingly, some particles with a fibrous habit that may be identified as asbestiform or simply as a cleavage fragment with a fiber-like habit were found in FEG-SEM micrographs (Fig. 9e and f). Analogously, nano-sized EMPs were found during dark-field STEM investigations (Fig. 15c and d). It is likely that these particles (fibrous particles and nano-sized EMPs) were produced by longitudinal splitting (Germine and Puffer, 2019), and they were not “native” asbestiform particles. We believe that these cleavage fragments must be taken into consideration when counting and measuring a given particle population since (1) the definition of EMPs includes both asbestiform and non-asbestiform morphologies (NIOSH, 2011); (2) the word “asbestos” does not have a definitive mineralogical significance (Case et al., 2011), being a commercial and regulatory definition (Williams et al., 2013), and there is a widespread “uncertainty and confusion about the specific nature of exposures described in many published studies” (NIOSH, 2011); and (3) the use of “trade names for mined asbestos minerals predated the development of rigorous scientific nomenclature” (NIOSH, 2011) and the non-scientific asbestos definition leads to a “taxonomic confusion and lack of standardized operating definitions of fibres” (Kane et al., 1996). Furthermore, most of the characteristics that define the asbestiform habit (and asbestos) identity might be considered controversial. As an example, a fibril is considered “an individual fiber of asbestos, generally a single crystal” (Veblen and Wylie, 1993 – glossary) or “the thinnest component (single crystal) of a fibre bundle” (Belluso et al., 2017); therefore, it would be impossible to apply the definition by Bailey et al. (2004), who described an asbestos fiber as “visible to the eye are bundles of individual crystal fibers known as fibrils [… ]”, because we would be unable to identify correctly a fibril (single crystal) by the naked eye or even by SEM, as a single crystal could be identified at this scale by TEM only. Along the same line, phase contrast microscopy (PCM), a method that is widely used for routine analysis, has been proven to have several limitations among which is the capability to distinguish and resolve very thin fibers and to differentiate different EMPs (NIOSH, 2011). One could even argue that defining a particle as flexible from an OM picture or an EM micrograph is impossible, since flexibility is a quantifiable mechanical property, whereas what is done operationally is a qualitative description of the presence of a “curved” particle. Probably, both the flexibility and the parallel arrangement of fibers in a native (unprocessed) sample are concepts that can be used by a geologist/mineralogist studying an outcrop, but they cannot be applied when describing a particle that has been collected from air or water, or has been extracted from biological material. In these cases, we cannot know how the native fibers actually occurred, and mechanical tests are not performed routinely by laboratories. In addition, there is no clear explanation of the mechanism by which a fiber that splits ceases to be carcinogenic (Germine and Puffer, 2019), while the fragments still have dimensions and physicochemical properties that can induce carcinogenesis (e.g., EMPs with certain dimensions and chemical composition). In conclusion, our aim is not to discuss the controversial nomenclature and definition related to asbestos and asbestiform materials, but we want to stress that it is crucial that the investigated EMP populations are represented in their entirety and not just as a portion of the population determined by the selection of a single magnification and thus a specific dimensional range of particles within the sample. We believe that the future development of our merged dataset method together with an operational multi-scale approach could lead to that goal.

Consistent with previously published data (Vigliaturo et al., 2018; Vigliaturo et al., 2020), the particle-habit frequency, dimensions, dimensional distribution, and assigned category by operational parameters (P) of the glaucophane studied here change when using different magnifications and instruments. Figure 16 demonstrates, for example, that the glaucophane samples investigated by SEM at a magnification of 200× exhibited proportions of crystal habits that were different from those observed when studied by SEM at a magnification of 2000× and from those studied by STEM (magnification 25 000×), even though the three samples were prepared identically. As expected, there is a difference in proportion of different habits between the particles studied by OM (sample not ground) and EM (sample ground for both SEM and TEM) (Fig. 16). The largest habit variations observed among all types of microscopy were found for bladed, platy, and fibrous particles, and to a lesser extent for acicular, lamellar, equant, and prismatic particles (Fig. 16). In addition, one should always consider that the definition of the habit might be biased by operator experience and background.

Using P1 (*L/w* ≥ 3), P2 (*L/w* ≥ 3 and *L*>5, i.e., EMP according to the NIOSH, 2011 definition), and P3 (fiber with *L/w* ≥ 3, *L* ≥ 5 μm, and *w* ≤ 3 μm, according to Belluso et al., 2017) as reference parameters, we observed that the STEM analyses at 25 000× returned very different results not only compared to the OM data but also compared to the SEM analyses conducted at 200× and 2000×, even though the latter two used the exact same ground material as the STEM analyses. Of all techniques, only the SEM data collected at 200× and 2000× revealed the presence of fibers with *L/w* ≥ 3, *L* ≥ 5 μm, and *w* ≤ 3 μm (P3). The tomographic investigation (Video Supplement – https://doi.org/10.5446/48657, Vigliaturo, 2020) showed that, in one orientation (Fig. 15a), the particle cannot be classified as a nano-sized EMP – according to the NIOSH (2011) and Oberdörster and Graham (2018) definitions – whereas in another orientation (Fig. 15b), the same particle has the dimensional characteristics of a nano-sized EMP.

To provide a visual representation of the change in particle size resulting from the use of different types of microscopy, the dimensional parameters (*L*, *w*, *L/w*, and *D*_ae_) of every individual particle (*n* = 100 for each magnification) were plotted in Fig. 17 against progressive particle number, whereby the particle numbers have been arranged from the largest to the smallest dimensions. This plot shows a similar trend for all considered dimensions except for *L/w*. We observed that changing the magnification from 200× (SEM) to 25 000× (STEM) led to an apparent decrease in particle dimensions. This decrease is not due to mechanical comminution since the sample preparation was identical for both the SEM and the STEM samples. Furthermore, the nano-sized portion (14 %) of the mineral particle population was detected only by the STEM (Table 4). The variation of the aver age particle dimensions (*w,L,D*_ae_) is about 1 order of magnitude per each order of magnitude change in magnification (Table 4 and Fig. 17).

These results have important implications for regulations in the area of environmental medicine, specifically in regard to which parts of the respiratory tract might be accessible to the particles. The location, where the particles might end up in the human respiratory tract, was determined by using the *D*_ae_ of each particle. As shown in Table 5, the number of particles in the inhalable, thoracic (PM_10_), respirable, and PM_2.5_ fractions is different for different microscopes and magnifications used. Specifically, the data obtained from the ground samples show that the SEM investigation performed at 200× underestimates the number of particles in the thoracic, respirable, and PM_2.5_ categories compared to that performed at 2000×. The same trend can be observed between the SEM investigation conducted at 2000× and the STEM investigation conducted at 25 000×. Overall, the current regulations for asbestos and EMPs may fail to completely describe the studied particle population, which would lead to an underestimation or overestimation of the percentage of certain habits and regulated particles, and thus distort the exposure-risk evaluation. This aspect does not dispute the validity of the current regulation itself, the sample-preparation methods, or measuring procedure of the particles but highlights the fact that using a single magnification is not sufficient to fully characterize a given particle population and detect all the EMPs that are present.

To overcome this problem, we have proposed a simple method of merging datasets collected at different magnifications, which provides a better and more representative picture of the overall characteristics of the studied particle populations. Usually, legislation and protocols to count, measure, and quantify asbestos and/or EMPs either suggest that the operator should work at a fixed magnification or an operational magnification is not specified (NIOSH, 1994a, b; European Commission Directive, 1999; WHO, 2000; ASTM, 2006; Council of the European Union, 2009; ASTM, 2015). In the following, we will compare the results obtained by our data-merging method with the dataset collected on the SEM at 2000×, which is a magnification that is typically suggested by different legislations (e.g., Italian legislation, Ministerial Decree, 1994) when measuring asbestos dimensions and quantifying the amount of asbestos present in bulk materials (Militello et al., 2019).

The first major difference is that the merged dataset contains information on all the operational parameters (P1, P2, and P3), thus documenting that SEM analyses conducted only at one magnification underestimate the percentages of particles for the operational parameters P2 and P3, which correspond to EMPs and fibers (as in Belluso et al., 2017), respectively (Fig. 16). The P2 particles amount to 13.00% of the SEM (2000×) data, whereas they represent 20.83% of the merged dataset. Similarly, the P3 particles represent 12.00% of the SEM (2000×) data but 14.35% of the merged dataset. On the other hand, the percentages of particles with *L/w* ≥ 3 are very similar for both datasets: 35.00% (SEM 2000×) and 33.33% (merged dataset).

A second difference is represented by the fact that the dimensional distributions obtained from the SEM (2000×) dataset exhibit a right-skewed normal distribution, whereas those obtained from our merged dataset can be described with a power law and cover a larger size range (Appendix D). The power-law distribution is mathematically equivalent to a fractal distribution (Carpinteri and Chiaia, 1997), identifying the fragmentation of our sample as a scale-invariant process, analogous to many processes observed in nature (Wylie, 1993). The dimensional distributions in the histogram charts obtained at 2000× for *L*, *w*, and *D*_ae_ always had their maximum in the second-smallest size category rather than in the smallest one (Appendix D). The lower relative frequency in the smallest size bin might be a consequence of (1) an arbitrary exclusion of particles with certain dimensional parameters, (2) a real decrease of the number of particles in the considered range, or (3) a portion of the particle population not being visible at the selected magnification (Wylie, 1993). This lower relative frequency in the smallest size bin of the 2000× dataset was not observed in any of the merged datasets (Appendix D), which suggests that, when performing single-magnification characterizations, it is likely a consequence of the use of a fixed magnification that does not allow for correct visualization of the smaller portion of the observed population. In our specific case, we think that the drop in the number of particles in the smallest bin of the OM and SEM data is due to two possible problems: (1) small particles occurring as clusters might not be recognized as individual objects but are grouped into larger particles because of spatial resolution limits and superimposition of particles, and/or (2) the presence of small crystallites hidden from the observer by larger particles (e.g., when lying below).

On the other hand, a drop in the number of particles in the smallest bin is not always observed by TEM since it virtually has an infinite resolution power, but it can occur because of the deposition of smaller crystallites on or along the copper grid (thus not visible) or because there is a real decrease of particles in the given dimensionality, since the material has reached its “minimum” possible dimension.

Our results document that the total percentage of EMPs and fibers (as in NIOSH, 2011; Belluso et al., 2017) is always underestimated when using a single magnification compared to the percentage obtained from the merged dataset, as proposed here. Furthermore, the information on the nano-sized fraction of the particle population is not present in the SEM dataset, whereas the merged dataset shows that this fraction represents 6.48% of the overall particle population (Table 4). This difference might be important in terms of potential health impacts, because the smaller particles are more likely taken up by non-phagocytic cells, i.e., alveolar epithelial cells and mesothelial cells (Le Bouffant et al., 1987; Gelzleichter et al., 1996; Nagai and Toyokuni, 2012).

In summary, the merged dataset is a “weighted” distribution of all the percentages determined by SEM (200×), SEM (2000×), and STEM (25 000×). Therefore, when taking the *D*_ae_ as the parameter that determines the eventual distribution of the particles within the lungs, the values obtained from the merged dataset return more realistic results (Table 5).

## 6 Conclusions

A detailed characterization of a glaucophane sample from northwestern Italy was conducted using different analytical techniques over different scales. A difference between the bulk and the grain-boundary Fe-valence state of individual amphibole particles was found, and the Δ*E* method to evaluate the valence state from EELS spectra in Al^3+^-containing amphiboles seems to be a more reliable approach than using the *L*_2,3_ white-line intensity-ratio method. The habit and dimensional distributions are different at different scales, and thus it is important that EMP regulations consider multiple magnifications when assessing potentially dangerous mineral particle populations and developing an operational method to count and measure particles. Our results indicate that when describing a population of potentially dangerous EMPs (both asbestiform and non-asbestiform), the use of a merged dataset, as proposed in this paper, is superior compared to the use of a fixed magnification, which is suggested by current legislations and protocols for asbestos (usually at 400×, 600×, 1000×, or 2000×). We conclude that the use of a merged dataset obtained from at least two datasets is fundamental in determining the real presence of, and thus potential exposure to, EMPs in a given sample.

## Supplementary Material

Supplement

Electron Tomography and 3D reconstruction of a glaucophane particle

## Figures and Tables

**Table 1.** ^57^Fe Mössbauer parameters for glaucophane. IS indicates isomer shift; QS indicates quadrupole splitting; HWHM indicates half width at half maximum. The standard deviations (*σ*_*n*−1_) are in parentheses.

Site	IS *δ* (mm/s)	QS Δ (mm/s)	HWHM Γ/2 (mm/s)	Area (%)
Fe^2+^*M*(1)	1.131(5)	2.835(9)	0.127(6)	35.3(9)
Fe^2+^*M*(3)	1.103(6)	2.499(16)	0.186(11)	25.7(1.4)
Fe^2+^*M*(2)	1.114(12)	2.200(14)	0.139(8)	19.9(1.2)
Fe^3+^	0.365(6)	0.475(11)	0.136(11)	17.1(9)
Fe^3+^	0.397(8)	0.81(2)	0.101(8)	2.0(1.3)

**Table 2.** Average EMPA data for the studied glaucophane. Chemical formula (in a.p.f.u.) calculated on the basis of 23 oxygen atoms.

Oxide	wt% (*n* = 35)	*σ*_(*n*−1)_
SiO_2_	58.45	0.59
TiO_2_	0.03	0.02
Al_2_O_3_	10.54	0.35
FeO	6.45	0.33
Fe_2_O_3_	1.79	–
MnO	0.06	0.03
MgO	11.91	0.28
CaO	1.17	0.43
Na_2_O	6.88	0.22
K_2_O	0.02	0.02
F	0.19	0.49
O_F_Cl	−0.08	0.01

Total	97.49	

Si	7.97	
Al	0.03	

∑ T site	8.00	
Al	1.66	
Fe^3+^	0.18	
Ti	0.003	
Mg	2.42	
Fe^2+^	0.74	
Mn	0.007	

∑ C site	5.00	
Ca	0.17	
Fe^2+^	0.00	
Na	1.83	

∑ B site	2.00	
Na	0.00	
K	0.003	

∑ A site	0.003	
F	0.08	
OH	1.92	
Mg/Mg+Fe	0.77	

**Table 3.** Calculated site occupancies for glaucophane.

Site	Cation	a.p.f.u.
*M*(1,3)	Fe^2+^	0.56
	Mg	2.42
	Fe^3+^	0.02

*M*(2)	Al	1.66
	Fe^3+^	0.16
	Fe^2+^	0.18

*M*(4)	Na	1.83
	Ca	0.17

**Table 4.** Summary of the descriptive statistics of glaucophane particles using different microscopy imaging techniques (*n* = 100 each, except for the merged dataset, where *n* = 216). The nano-sized (<100 nm) component is determined using *D*_ae_ as reference.

	Optical (10×)				
	*L* (μm)	*w* (μm)	*L/w*	*D*_ae_ (μm)	Nano-sized component

Mean	155.55	62.32	2.66	131.67	0.00%
σ(*n*−1)	112.18	43.21	1.16	89.93	
Max	654.87	249.25	7.33	485.24	
Min	14.53	8.59	1.05	16.34	

	SEM (200×)				
	*L* (μm)	*w* (μm)	*L/w*	*D*_ae_ (μm)	Nano-sized component

Mean	15.25	6.22	3.32	12.67	0.00%
σ(*n*−1)	10.46	5.16	2.70	9.53	
Max	59.56	28.49	16.14	58.91	
Min	2.39	0.99	1.02	2.16	

	SEM (2000×)				
	*L* (μm)	*w* (μm)	*L/w*	*D*_ae_ (μm)	Nano-sized component

Mean	2.93	1.30	3.01	2.51	0.00%
σ(*n*−1)	2.30	1.23	3.50	2.07	
Max	11.71	8.79	33.13	13.26	
Min	0.41	0.14	1.02	0.36	

	STEM (25 000×)				
	*L* (μm)	*w* (μm)	*L/w*	*D*_ae_ (μm)	Nano-sized component

Mean	0.31	0.15	2.30	0.29	14.00%
σ(*n*−1)	0.28	0.11	1.98	0.21	
Max	1.93	0.54	17.23	1.15	
Min	0.05	0.02	1.06	0.04	

	Merged			
	*L* (μm)	*w* (μm)	*L/w*	*D*_ae_ (μm)	Nano-sized component

Mean	6.69	2.65	3.17	5.54	6.48%
σ(*n*−1)	10.13	4.60	3.21	8.82	
Max	59.56	28.49	33.13	58.91	
Min	0.05	0.02	1.02	0.04	

**Table 5.** Percentage of particles that belong to a specific particle-size category based on their *D*_ae_, which was determined by using different microscopy imaging techniques.

	Optical 10×(not ground)	SEM 200×(ground)	SEM 2000×(ground)	TEM 25000×(ground)	Merged
Inhalable (<100 μm)	43.00%	100.00%	100.00%	100.00%	100.00%
PM10 (equivalent to thoracic)	0.00%	49.00%	99.00%	100.00%	81.02%
Respirable (<4 μm)	0.00%	10.00%	86.00%	100.00%	65.74%
PM_2.5_	0.00%	1.00%	66.00%	100.00%	55.09%

## Data Availability

All the data of this research are presented in the manuscript and additional material is uploaded as a Supplement.

## Appendix D: Additional dimensional information

These sections display the histograms obtained from our dataset by imposing 20 bins arbitrarily. The data are the same as those shown in the main text (Fig. 17). All data are listed in units of μm, except for *L/w*, which is dimensionless.

The blue charts correspond to OM data (10×), the orange to SEM data (200×), the yellow to SEM data (2000×), and the grey to TEM data (25 000×). Furthermore, the histograms obtained from the merged datasets (SEM 200×; SEM 2000×; STEM 25 000×) are shown in red.

Data for length (*L*)

Data for width (*w*)

Data for aspect ratio (*L/w*)

Data for aerodynamic equivalent diameter (*D*_ae_)

Data for the merged datasets
