# Supplementary material for: Multi-scale characterization of glaucophane from Chiavolino (Biella, Italy): implications for international regulations on elongate mineral particles
Source: Eur J Mineral. Author manuscript; Available in PMC 2021 Apr 9. (PMC8034610; doi:10.5194/ejm-33-77-2021)
Supplement: Supplement [file NIHMS1680470-supplement-Supplement.zip › ejm-33-77-2021-supplement-title-page.pdf]

Supplement of Eur. J. Mineral., 33, 77–112, 2021
https://doi.org/10.5194/ejm-33-77-2021-supplement
© Author(s) 2021. This work is distributed under
the Creative Commons Attribution 4.0 License.

European Journal of Mineralogy
Open Access

Supplement of

Multi-scale characterization of glaucophane from Chiavolino (Biella, Italy): implications for international regulations on elongate mineral particles

Ruggero Vigliaturo et al.

Correspondence to: Ruggero Vigliaturo (ruggero.vigliaturo@gmail.com)

- ejm-33-77-2021-supplement-title-page.pdf
- Supplementary merged dataset.xlsx

The copyright of individual parts of the supplement might differ from the CC BY 4.0 License.
